# Mapping frontoinsular cortex from diffusion microstructure

**DOI:** 10.1093/cercor/bhac237

**Published:** 2022-06-27

**Authors:** Ryan P Cabeen, Arthur W Toga, John M Allman

**Affiliations:** Laboratory of Neuro Imaging, USC Stevens Institute for Neuroimaging and Informatics, Keck School of Medicine of USC, University of Southern California, Los Angeles, CA 90033, United States; Laboratory of Neuro Imaging, USC Stevens Institute for Neuroimaging and Informatics, Keck School of Medicine of USC, University of Southern California, Los Angeles, CA 90033, United States; Division of Biology and Biological Engineering, California Institute of Technology, Pasadena, CA 91125, United States

**Keywords:** frontoinsular cortex, anterior agranular insular cortex, diffusion MRI, decision-making, social behavior

## Abstract

We developed a novel method for mapping the location, surface area, thickness, and volume of frontoinsular cortex (FI) using structural and diffusion magnetic resonance imaging. FI lies in the ventral part of anterior insular cortex and is characterized by its distinctive population von Economo neurons (VENs). Functional neuroimaging studies have revealed its involvement in affective processing, and histopathology has implicated VEN loss in behavioral-variant frontotemporal dementia and chronic alcoholism; however, structural neuroimaging of FI has been relatively limited. We delineated FI by jointly modeling cortical surface geometry and its coincident diffusion microstructure parameters. We found that neurite orientation dispersion in cortical gray matter can be used to map FI in specific individuals, and the derived measures reflect a range of behavioral factors in young adults from the Human Connectome Project (*N*=1052). FI volume was larger in the left hemisphere than the right (31%), and the percentage volume of FI was larger in women than men (15.3%). FI volume was associated with measures of decision-making (delay discounting, substance abuse), emotion (negative intrusive thinking and perception of hostility), and social behavior (theory of mind and working memory for faces). The common denominator is that larger FI size is related to greater self-control and social awareness.

## Introduction

The human cerebral cortex is the outermost layer of the cerebrum with a macroscopic convoluted structure and microscopic cytoarchitecture consisting of laminar structure differentiated by cell morphology, density, and connections (Nieuwenhuys et al. 2007). The pattern of these cytoarchitectural features vary across the cortex and often change in relation to folding patterns and gyral shape, providing one way to localize cortical areas, or parcels (Van Essen et al. 2013). Because the structural integrity of the cortex underlies both healthy and abnormal brain function, image analysis tools have been developed to create models of the cortex from structural magnetic resonance imaging (MRI) data (Fischl 2012, Shattuck and Leahy, 2002, Zhong et al. 2010). Such approaches create 3D geometric models that may be spatially normalized using surface-based registration to transfer cortical labels from brain atlases to individual subject data (Desikan et al. 2006, Glasser et al. 2013, Klein and Tourville, 2012), most often on the basis of cortical folding patterns or functional activation (Blumensath et al. 2013, Van Essen et al. 2013), and these surface-based analyses provide superior spatial localization compared with more traditional voxel-based analysis (Coalson et al. 2018). This further enables the morphometric characterization of the cortex using geometric surface measures such as cortical thickness, volume, and surface area (Cardinale et al. 2014, Tustison et al. 2014); however, morphometry-based parcellation may not reflect a variety of important microstructure features of cortical cytoarchitecture, such as dendritic complexity, cell density, myelination, etc.

Diffusion MRI has emerged as a tool for probing tissue microstructure features, made possible through a combination of multi-shell diffusion encoding sequences and mathematical modeling techniques (Alexander et al. 2019). While traditionally employed to examine white matter, it is now being used to measure microstructural features of gray matter as well (Nazeri et al. 2020). Fukutomi et al. (2018) demonstrated the feasibility of investigating such features across the entire cortex by combining diffusion and structural MRI modalities. Several groups have recently applied this approach to study group level features of human behavior, Alzheimer’s disease, substance use, life satisfaction, and others (Baxi et al. 2020, Cabeen et al. 2020, Caron et al. 2020, Genç et al. 2018, Nazeri et al. 2015, Schmitz et al. 2019, Torso et al., Vogt et al. 2020). We have been investigating such approaches for specifically characterizing frontoinsular cortex (FI) (Cabeen et al. 2020, 2021), and in this paper we introduce, evaluate, and apply a technique for mapping the location, volume, and surface area of FI, and we quantitatively investigate how FI volume relates to a variety of behavioral variables in young adults. FI is already known to contribute to social decision-making and contains a distinctive population of large bipolar cells, the von Economo neurons or VENs (Allman et al. 2010), which degenerate in the behavioral variant of frontotemporal dementia (bvFTD) and in alcoholism (Senatorov et al. 2015). VEN loss is correlated with the severity of sociocognitive deficits in bvFTD such as increased impulsivity and loss of empathy (Kim et al. 2012, Pasquini et al. 2020). Using region-of-interest (ROI) analysis, we previously reported that the microstructure of FI, as measured in diffusion MRI by the orientation dispersion index (ODI) and by fractional anisotropy (FA), is influenced by the degree of life satisfaction, the use of cannabis, and negative intrusive thinking, based on the Human Connectome Project (HCP) data (Cabeen et al. 2020. 2021). ODI is a negative measure and FA is a positive measure of the spatial alignment of cell membranes and other microstructural components. In FI, the microstructure quantified in these ways increases with greater life satisfaction and decreases with cannabis use and negative intrusive thinking (Cabeen et al. 2020, 2021). Menon et al.  (2020) have also examined cortical microstructure as measured with the diffusion return-to-origin-probability noting distinctive features of insular cortex as well.

In conducting these earlier studies, we noticed that FI tended to have lower ODI and higher FA than surrounding cortex, which motivated us to examine whether the extent of FI could be mapped by using these diffusion imaging parameters projected onto cortical surface models. In these previous studies, we applied atlas-based ROI analysis to localize FI, using either voxel-based and surface-based registration. Such ROI analyses depend heavily on the quality of image or surface registration, which is most often based on brain shape, rather than microstructure characteristics. In the present paper, we instead propose and investigate an approach that delineates FI by thresholding surface-defined cortical microstructure parameters, which can hypothetically segment FI based on cytoarchitecture features, as opposed to surface morphometry. Our experiments examine the optimal threshold parameters for FI delineation, compare our segmentations with a priori manual labeling, measure the scan–rescan reliability of our approach, and analyze twin and nontwin siblings (NT) to determine heritability. We further asked whether FI volumes derived from these maps for individual subjects differed between the left versus right hemispheres, between male and female participants, and in relation to a suite of demographic and behavioral characteristics that might be associated with FI processing. Our data were obtained from the Human Connectome Project (*N*=1052), which provides a large cohort of typical young adults scanned with a high-quality diffusion MRI sequence suitable for multi-modal analysis with cortical surface modeling. In the following sections, we describe our method and report results that together indicate FI volumes estimated using our approach are reliable, heritable, and sensitive to behavioral measures of decision-making, emotion, and social behavior.

## Methods

Our experiments use datasets and some preprocessing steps that are similar to our previous studies (Cabeen et al. 2020, 2021); these are briefly described again in the following subsections. We further describe the novel components of our pipeline for mapping the location, volume, and surface area of FI, as well as the statistical approach taken in our experiments.

### Participants and datasets

Data for our experiments were acquired from participants as part of the Young Adult HCP (Van Essen et al. 2019). We performed a multi-modal analysis that included both T$_1$-weighted (T1wMRI) and diffusion-weighted MRI (dwMRI) data, and in total, we included 1052 participants (571 female, 481 male) with scans that passed quality control and completed image processing. With approval from the Institutional Review Boards of the University of Southern California and the California Institute of Technology, we accessed and analyzed demographic and behavioral data from the restricted data release. In total, we examined 115 demographic and behavioral variables, which we chose as plausible candidates to reflect variation in FI structure. In particular, this included measures from the NIH Toolbox Emotion battery (emotion recognition, psychological well-being, social relationships), the NIH Toolbox Cognition battery (self-regulation/impulsivity, executive functioning, memory, working memory, measures of life function from the Achenbach Adult Self-Report (ASR), performance from the in-scanner tasks (social cognition, language, and working memory), theory of mind (TOM), psychiatric history, substance use (smoking, alcohol, Tetrahydrocannabinol (THC)), olfaction, taste, and pain. Unlike some other measures, THC exposure is not self-reported, but rather obtained from a chemical biospecimen test. Several of these measures were part of a collection of related measures, e.g. delay discounting across a range of monetary amounts and time periods, working memory performance, and cognitive performance; in these cases, we also computed a single general factor to summarize the group by computing the scores associated with the first principal component. We have summarized all of the behavior variables we investigated in Table 1 and in the Supplementary Material.

**Table 1:** Behavioral variables examined in our experiments.

Category	Description
**Cognition**—Executive	Picture Sequence Memory Test, Dimensional Card Sorting Test, Flanker Test, Processing Speed Test
**Cognition**—Intelligence	Cognition Scores for Fluid Ability, Early Childhood Composite, Crystallized Composite & Total Composite
**Cognition**—Memory	List Sorting, Working memory task in the scanner: accuracy and reaction time for 0/2-back with multiple stimuli types
**Cognition**—Language	Reading English Test, Picture Vocabulary Test, Language task assessed in scanner, including overall performance, story, and math measures
**Emotion**—Recognition	Measures from the Penn Emotion Recognition Test (ER-40), including correct responses, average response time, and scores for anger, fear, happiness, sadness, and neural.
**Emotion**—Matching	An in-scanner emotion task matching faces and shapes with measures of accuracy and reaction time for each
**Emotion**—Perception	Self perceived personal negative affect including sadness, anger (affect, physical & hostility), fear (affect and somatic)
**Emotion**—Well-being	Positive Affect, Life Satisfaction, Meaning & Purpose, Perceived Stress, Self-Efficacy
**Emotion**—Psychiatric	18 measures from the Achenbach ASR. 5 measures from the Semi-Structured Assessment for the Genetics Alcoholism
**Social**—Relationships	6 measures reflecting Social Support, Companionship, Social Distress, Positive Social Development
**Social**—TOM	5 measures of TOM, assessed in scanner, including reaction time and percentage labeled in each group
**Decisions**—Self control	Delay discounting—subjective value assessed for $200 and $40k at 1 and 6 months and 1, 3, 5, and 10 years
**Decisions**—Drinking	Total number of drinks in past week, number of days drank in past week
**Decisions**—Smoking	Total times used tobacco in past week, times used tobacco in past week, days using tobacco in past week
**Decisions**—Drug use	Biospecimen test for a recent positive THC chemical test, self-reported paternal drug and alcohol problems
**Sensory**—Perception	Odor, Taste, Pain interference

### Image acquisition and preprocessing

The data were collected using the imaging protocol designed for the HCP, which we briefly summarize here. The T1wMRI and dwMRI data from the HCP were collected on a Connectome Siemens 3 Tesla Skyra scanner using a 32-channel head coil (Glasser et al. 2016, Van Essen et al. 2019). The T1wMRIs were acquired using a 3D MPRAGE sequence with 0.7-mm isotropic resolution (field of view (FOV) = 224 mm, matrix = 320, 256 sagittal slices in a single slab), repetition time (TR) = 2400 ms, echo time (TE) = 2.14 ms, inversion time (TI) = 1000 ms, flip angle = 8$^{\circ }$, bandwidth = 210 Hz per pixel, echo spacing = 7.6 ms, and phase encoding undersampling factor GRAPPA = 2.10%. dwMRIs were collected with a single-shot 2D spin-echo echo-planar imaging (EPI) acquisition with a multi-band factor of 3, 1.25-mm isotropic voxels with FOV phase encoding (PE) by readout = 210 $\times $ 180; matrix size PE by readout = 144 $\times $ 168; 111 interleaved slices without gap; left–right and right–left phase encoding; flip angles = 78$^{\circ }$ and 160$^{\circ }$. For each phase encoding direction, the diffusion sampling scheme consisted of 18 baseline scans and 270 diffusion-weighted scans acquired using single diffusion encoding across 3 shells with *b* = 1000, 2000, and 3000 s/mm$^2$; all dwMRI scans had TE = 89 ms and TR = 5.5 s. Each shell included 192 data points representing 90 diffusion gradient directions and 6 *b* = 0 shells acquired twice resulting in 270 noncollinear directions for each PE. Total acquisition time was approximately 54 min (6 segments of 9 min each). dwMRI data were preprocessed with the HCP workflow (Sotiropoulos et al. 2013). This included the sophisticated approach for correction of artifact due to motion and eddy-current and susceptibility-induced geometric distortion in FSL EDDY. Using an additional set of diffusion MRI scans collected with reversed phase encoding, this scheme estimates and corrects for the off-resonance field and subject head motion using a Gaussian process framework for robust nonparametric interpolation of the dwMRI signal. This is a crucial step for mapping cortical microstructure, as surface-based mapping of microstructure parameters requires accurate alignment between T1wMRI and dwMRI data, which can be otherwise disturbed by susceptibility-induced geometric distortion.

### Image analysis

**Fig. 1:**
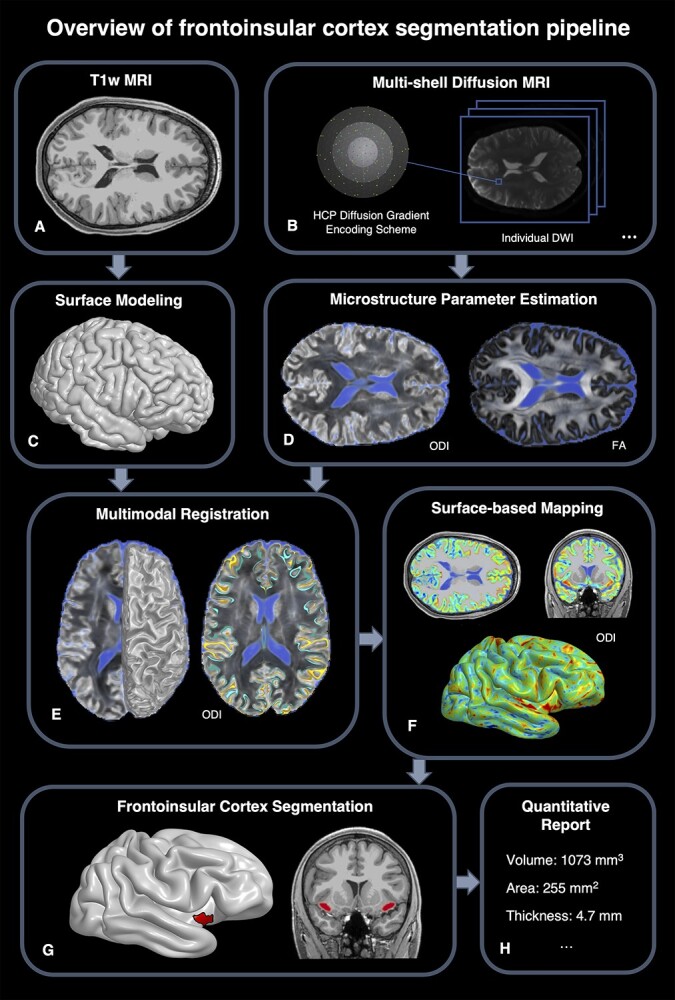
An overview of our image analysis pipeline, which combines surface-based modeling of T1wMRI data (A,D) with microstructure modeling of dwMRI data (B,D) to segment (E,F,G) and quantify FI volume, thickness, and surface area (H).

Building on our previous work (Cabeen et al. 2020, 2021), our image analysis took a multimodal approach that combines microstructure modeling with dwMRI data and cortical surface modeling from T1w MRI data. Our image analysis pipeline is illustrated in Fig. 1, and our workflow was implemented using the LONI Pipeline (Dinov et al. 2009) with components including the Quantitative Imaging Toolkit (QIT) (Cabeen et al. 2018), Freesurfer (Fischl 2012), FSL (Jenkinson et al. 2012), and ANTs (Avants et al. 2008).

The dwMRI data were denoised using a nonlocal means filter and microstructure parameters were obtained using 2 multi-shell modeling approaches. First, we performed neurite orientation dispersion and density imaging (Zhang et al. 2012) and estimated its parameters using a nonlinear fitting approach accelerated using the spherical mean technique (Cabeen et al. 2019), resulting in volumetric maps of the ODI and neurite density index (NDI). Because our experiments look specifically at gray matter, we used a parallel diffusivity of 1.1 $\times $ 10$^{-3}$ mm$^2$/s, which is an optimized value obtained from previous work (Fukutomi et al. 2018). NDI is meant to depict the proportion of neurite volume relative to the total cellular volume, whereas ODI is meant to separately depict neurite orientational heterogeneity. We also estimated diffusion tensor imaging (Basser and Jones 2002) parameters using weighted linear least squares fitting with free-water elimination with a fixed diffusivity of 3.0 $\times $ 10$^{-3}$ mm$^2/s$ in the isotropic compartment using the nonlinear least squares approach of Hoy et al. (2014), resulting in volumetric maps of FA and mean diffusivity. We used these 2 distinct diffusion modeling approaches to understand how they relate, because they are both widely used and useful points of reference. An important piece, however, is that they both include a free-water compartment, which can reduce partial volume effects due to subvoxel mixing of cerebrospinal fluid and gray matter tissue. Generally speaking, ODI and FA may reflect similar tissue properties, but they are inversely related, as lower neurite dispersion is reflected by an increase in tensor anisotropy.

We processed T1wMRI data using Freesurfer version 5.3.0-HCP to create 3D geometric models for the inner and outer cortical surface. The cortical models were aligned with dwMRI data using ANTs to compute the optimal rigid transform between the T1wMRI and average baseline diffusion image, and microstructure parameters maps were spatially normalized in the higher-resolution T1wMRI space using tricubic interpolation. To estimate cortical microstructure, we took a similar approach to Fukutomi et al. (2018) and used a statistical procedure to refine the alignment of the cortical surface to better match the tissue boundaries in the diffusion scan, in the event that any subtle geometric distortions remain after artifact correction. Briefly, for each subject, we computed the weighted average microstructure parameters in each vertex of the Freesurfer cortical surface, which consisted of 2 stages. In the 1st stage, the midpoint between the pial and white matter surfaces was computed and 15 sampling points were equally spaced between them. For a given microstructure parameter, the values were measured at each of the sampling point and subsequently weighted using a Gaussian function centered at the midpoint with a weight of one, with an SD that gives a weighting of 0.05 at each of the inner and outer cortical boundaries. This weighting scheme is designed to carefully isolate gray matter voxels by reducing the influence of voxels that are closer to tissue boundaries, which are more prone to partial volume effects. In the 2nd stage, statistically robust estimates the mean and SD microstructure parameters were computed, where outlier values were detected and excluded using a z-score threshold of 3.0; then, the average value was recomputed from points that remained after outlier rejection. This process is implemented in the QIT module named **VolumeSampleCortex**, and the final result is a collection of cortical microstructure parameters estimated at each surface vertex.

We subsequently performed a novel step for segmenting FI cortex. We first resampled the microstructure parameters using the spherical coordinates defined using Freesurfer’s surface-based atlas registration. We computed vertex-wise estimates of surface area, thickness, and volume using the approach of Winkler et al. (2012), implemented in QIT. This approach uses a more precise procedure for computing these morphometric surface features, particularly in areas of high curvature at the crests of gyri and depths of sulcae. Using the population average Freesurfer mesh coordinates, we then manually defined a restriction surface mask using QIT (shown in Supplementary Materials). This restriction surface mask was a roughly circular region centered around the anterior insula, and it is meant to limit the cortical extent that may possibly be included in our FI segmentation. We then applied the following steps to parcellate FI, which are implemented in the QIT module named **MeshAttrParcel**. First, the surface microstructure parameters were filtered on the surface using Laplacian smoothing in 2 iterations with $\lambda = 0.3$. Then, a threshold was applied to vertices within the restriction mask to compute an FI surface mask, followed by a mode-filter regularization step and by extracting the largest connected component. Finally, we computed the surface area, average thickness, and total volume of cortex in the resulting FI surface mask. These steps were performed separately for each hemisphere and separately using ODI and FA. The direction of the thresholding was opposite for FA and ODI, so when performing ODI-based segmentation, vertices below the threshold were included, but when using FA-based segmentation, vertices above the threshold were included. We also computed percentage estimates of surface area and volume, which were obtained by dividing by the FI surface area and volume and estimated by analogous whole cortex estimates, respectively. These steps are illustrated in Fig. S1 in the Supplementary Material.

### Statistical analysis

Our statistical analysis explored the optimal threshold for FI delineation, investigated the reliability of our FI maps with a test–retest analysis, measured heritability with twin data, determined differences between left and right hemispheres as well as males and females, and finally, examined the relationship between a suite of behavioral variables related cognition, decision-making, emotion processing, and social functioning.

Our statistical analysis was implemented using R 3.3.3, plots were created using ggplot 3.2.1 (Wickham 2017), and tables were created using stargazer 5.2.2 (Hlavac, 2013). 3D visualizations of statistical maps overlaid on brain anatomy were created using QIT. The details of our statistical tests are described as follows.

Our experiments were performed for both ODI- and FA-based FI mapping, so as to evaluate their relative performance. We also measured whole cortical volume and the volume of agranular anterior insular cortex (AAIC) as defined using the HCP multimodal parcellation (HCP-MMP-1.0) (Glasser et al. 2013). These are both measures based solely on Freesurfer analysis of T1w MRI data and were chosen to provide a baseline for comparison.

#### Threshold optimization

Our optimization of the delineation threshold involved a parameter sweep across candidate thresholds from 0 to 1 with increments of 0.025. We extracted FI volumes for the entire HCP cohort (*N*=1052) and measured descriptive statistics of the distribution of FI volumes across the cohort for each candidate threshold. Our primary goal was to minimize the skewness of the distribution, so as to identify a threshold that produces a near Gaussian distribution of FI volumes and is minimally biased. We repeated this experiment for both ODI- and FA-based segmentation and obtained optimal thresholds for each. We also created population-average maps of FI on both the MNI atlas and an average cortical surface in MNI space.

#### Reliability analysis

We performed a reliability experiment, which used a secondary test–retest dataset from the HCP with 2 scans collected for 44 individuals. We measured reliability of FI volume, surface area, and thickness using the intraclass correlation (ICC) and the coefficient of variation (CoV), for each of the FA- and ODI-based segmentation results. Given a mean parameter value $\mu $, a within-session variance $\sigma _{within}$, and a between-subjects variance $\sigma _{between}$, the ICC was computed by $(\sigma _{between} - \sigma _{within}) / (\sigma _{between} + \sigma _{within})$, and the CoV was computed by $\sigma _{within} / \mu $.

#### Demographic variables

To examine FI lateralization, we estimated the volume, surface area, and thickness of FI for each hemisphere and computed descriptive statistics, and we statistically tested for left–right differences with Student’s *t*-test. We made a similar comparison between participants who self-identified as male and female. Next, we computed the heritability of each FI parameter in a subset of the HCP cohort that were siblings. We identified sibling pairs by matching parental identifiers and then separating based on zygosity to obtain 3 groups: monozygotic twins (MZ), dizygotic twins (DZ), and NT. We computed the correlation between MZ ($r_{MZ}$), DZ ($r_{DZ}$), and NT ($r_{NT}$) siblings, and obtained the heritability estimates using Falconer’s formula, $H^{2}_{b} = 2 (r_{MZ} - r_{DZ})$, (Falconer and Mackay 1996).

#### Behavioral variables

We examined 115 behavioral variables that were chosen for possible involvement with FI processing, including cognition, emotion, decision-making, and social functioning (Table 1). We used multiple linear regression modeling to examine the relationship between FI volume (averaged between left and right hemispheres) and each variable with the following approach. All continuous model parameters were normalized to zero-mean and unit-variance to allow their regression coefficients to be reported in standardized units. We excluded outliers using Tukey’s procedure, in which high and low cutoffs were determined by 1.5 times the interquartile range beyond the low and high quartiles, computed using the entire cohort. We retained the R$^2$ coefficient of determination of the model, and the statistical outcomes of each subject variable, including the standardized regression coefficient $\beta $, *t*-value, standard error, and *P*-value. We estimated linear regression models in a stepwise fashion to test covariates that included sex, age, total intracranial volume, total brain surface area, and whole-brain averages of ODI and FA. We corrected for multiple comparisons with the false discovery rate (FDR) procedure of Benjamini and Hochberg (1995) and determined statistical significance with an FDR *q*-value threshold of 0.05. We also computed the change in Bayesian information criterion ($\Delta $BIC) from adding each behavioral parameter to the model, which offers an additional evidence for the importance of a given effect, where a larger positive $\Delta $BIC indicates stronger evidence, which zero or lower indicates a lack of evidence.

## Results

Representative example results from 3 individuals are shown in Fig. 3; these cases were randomly chosen to represent the low quartile, median, and high quartile ranges of FI volumes. Population average results are shown in Fig. 4, including an FI segmentation probability map and average cortical microstructure parameters. We created contour plots to show the shape and size of the FI probability maps, and these are shown in Fig. 5. We also created 3D visualizations to show the relationship between the location of FI and the underlying diffusion principal orientations (which were not used for segmentation purposes). Unless otherwise stated, our results focused on ODI-based FI segmentation, but we have also included analogous results tables and plots for FA-based segmentation in the Supplementary Materials. We generally found that ODI-based mapping was more sensitive than FA-based mapping. We further found that microstructure-based mapping of FI (with either ODI or FA) was much more sensitive that Freesurfer-based mapping of AAIC volume. Next, we review the specific results in detail.

**Fig. 2:**
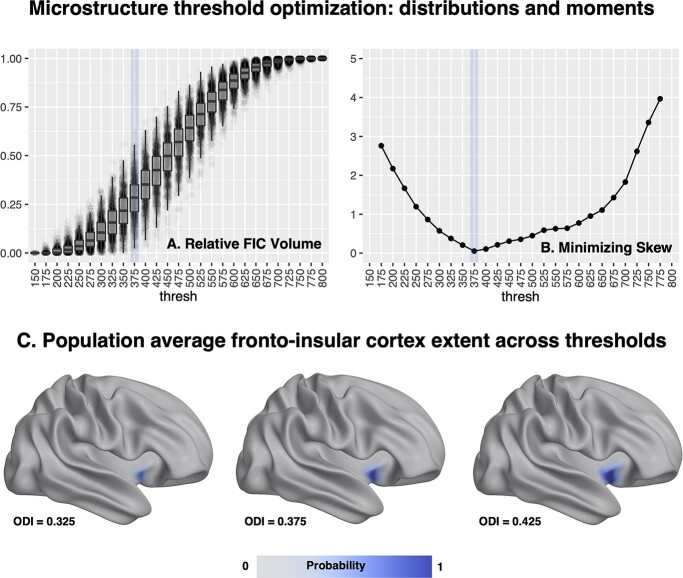
Visualizations of our steps to optimize the threshold for delineating FI using ODI. Panel A shows the distribution of FI volumes obtained across a range of thresholds. We computed descriptive statistics for the skewness (Panel B). The desirable ranges of high variance and low skewness are highlighted in blue. The bottom row (Panel C) shows visualizations of the population average FI map for three thresholds around the the optimal threshold of 0.375. An analogous plot for FA-based mapping can be found in the Supplementary Materials.

**Fig. 3:**
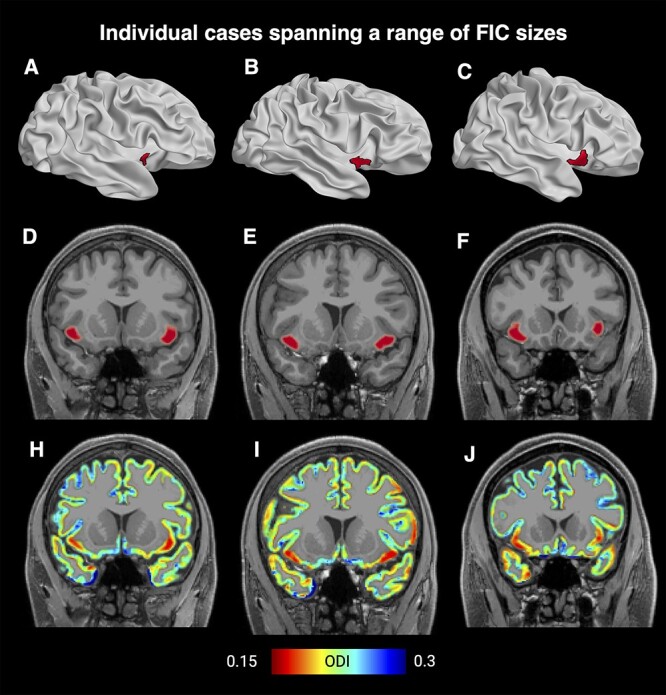
Visualizations showing randomly selected individuals that represent small,medium, and large FI maps based on ODI. Shown are inflated cortical surface models with FI highlighted in red (A, B, C), coronal sections with a red FI overlay (D, E, F), and coronal section with an ODI gray matter overlay (H, I, J). The individuals were selected in a 2-step process, where we first selected 10 individuals who were closest to each of the low quartile, median, and high quartiles, and we then selected a random individual from each of those subsets. An analogous plot for FA-based mapping can be found in the Supplementary Materials.

**Fig. 4:**
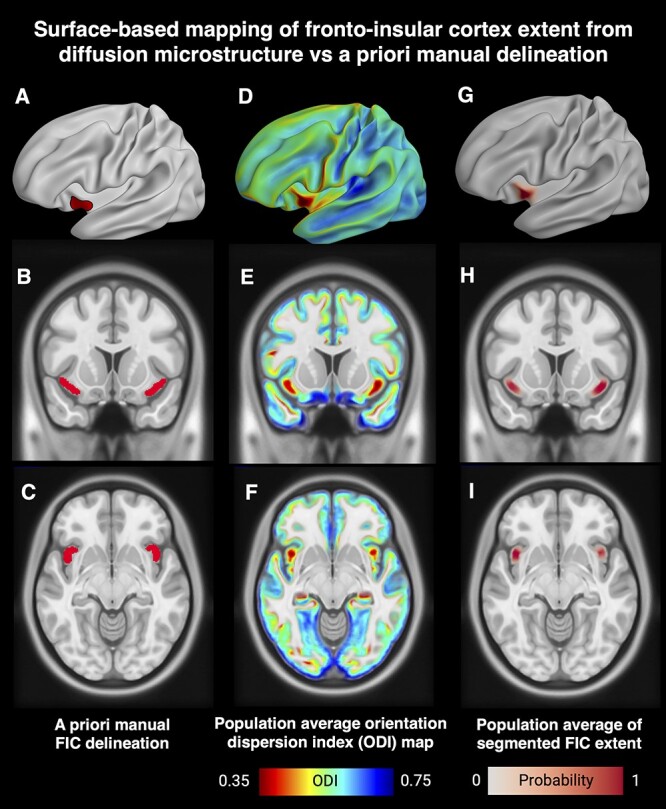
Visualizations showing population average FI mapping results using ODI. The first column shows an a priori manual delineation made for our previous studies (A, B, C). The middle column shows the HCP average ODI data on an inflated cortical surface and 2 image slices (D, E, F). The right column shows the HCP average FI segmentation on an inflated cortical surface model and 2 image slices (G, H, I). An analogous plot for FA-based mapping can be found in the Supplementary Materials.

**Fig. 5:**
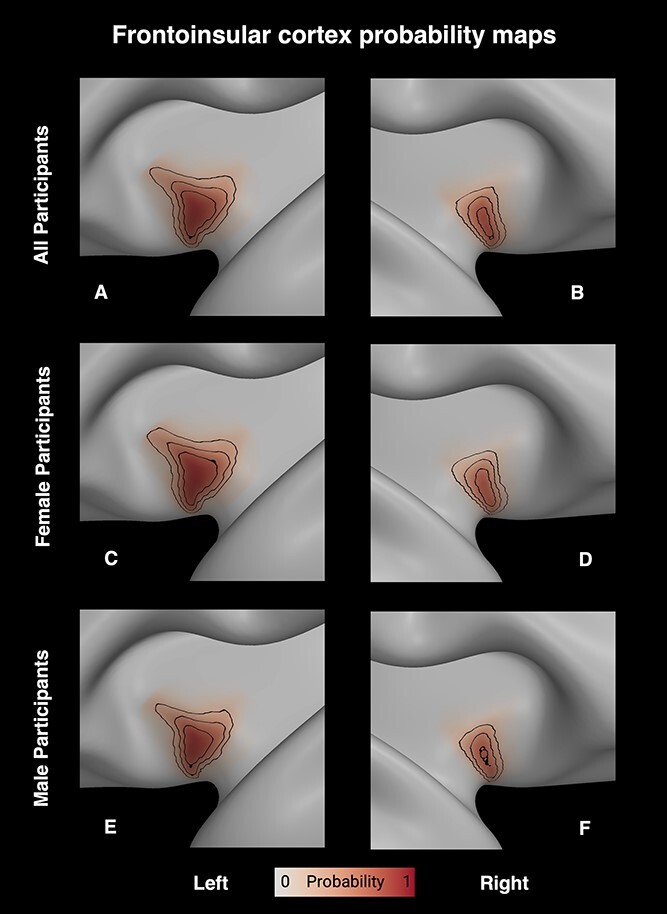
Visualizations of the HCP average ODI-based FI maps with contour lines drawn at the 25%, 50%, and 75% probability values in each case. The top row shows the left and right hemisphere averages (A, B), and the middle and bottom rows show the averages for female (C, D) and male (E, F) participants, respectively.

**Fig. 6:**
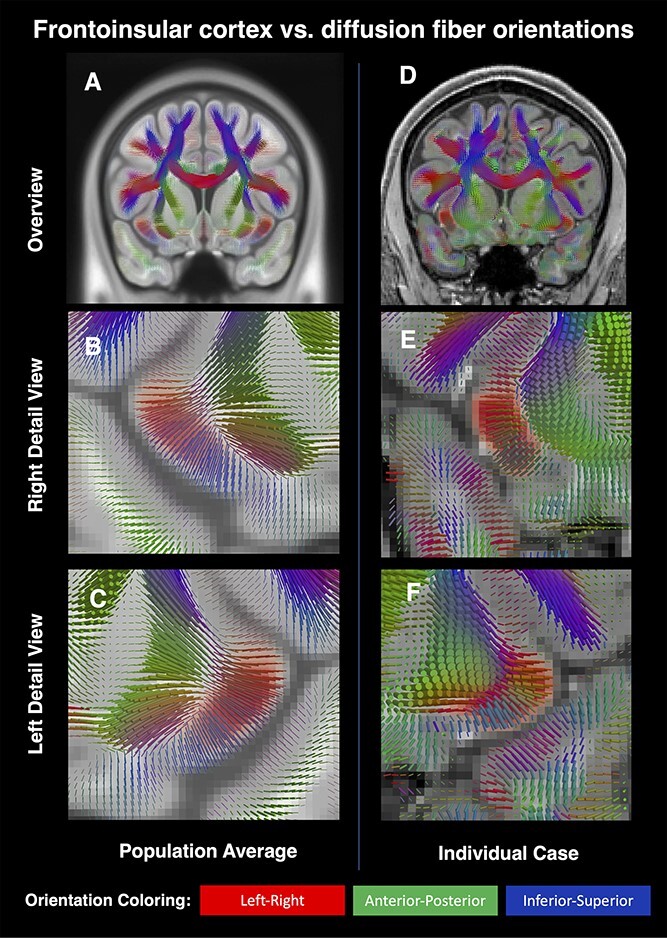
Visualizations showing the relationship between our FI maps (red) and principal diffusion orientations (direction-color cylinders). The left column shows HCP average orientations and and FI probability (A, B, C), whereas the right column shows similar data in a randomly chosen individual (D, E, F).

### Threshold optimization

Our analysis of FI segmentation thresholds focused on the skewness as distributional measures of interest. Plots of our results are shown in Fig. 2 and also in the Supplementary Material. We found that an ODI threshold of 0.375 had the minimal skewness. In a similar way, we found the optimal threshold for FA-based delineation was 0.180. After running our analysis, we also tested threshold values one increment below and above our optimum to ensure the stability of our findings. We used these optimal thresholds in the experiments presented in the remainder of the paper. The various FI metrics produced by the pipeline at this optimal threshold are summarized in the Supplementary Material.

### Reliability

**Fig. 7:**
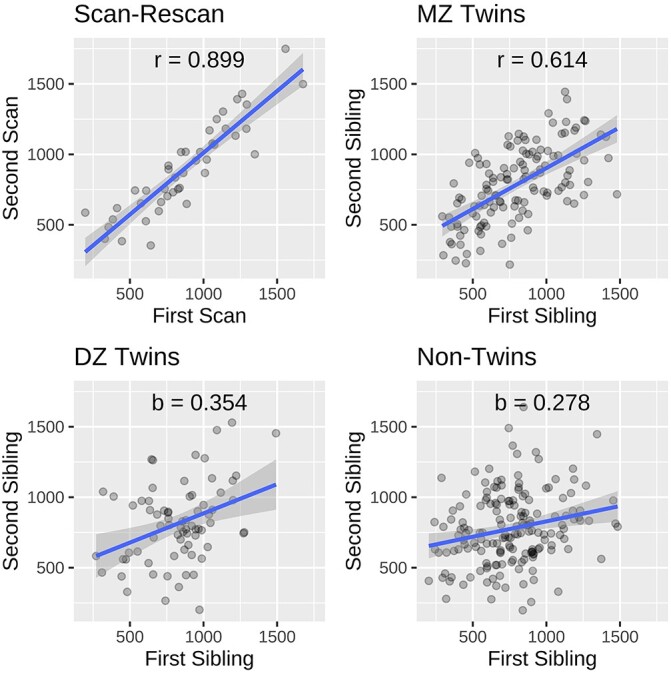
Plots showing variation in ODI-based FI volume across repeated scans of the same individual (A) and pairs of siblings, including MZ (B), DZ (C), and NT (D). The plots show a general trend where greater genetic similarity corresponds with higher similarity between FI volume estimates. An analogous plot for FA-based mapping can be found in the Supplementary Materials.

Our analysis of test–retest data showed that, using ODI-based segmentation, FI volume had ICC = 0.91 and CoV = 8.0%, FI surface area had ICC = 0.89 and CoV = 8.3%, and FI mean thickness had ICC = 0.31 and CoV = 2.5%. Using FA-based segmentation, FI volume had ICC = 0.84 and CoV = 10.8%, FI surface area had ICC = 0.84 and CoV = 11.3%, and FI mean thickness had ICC = 0.41 and CoV = 2.7%. Thus, ODI-based segmentation tended to produce more reliable estimates than FA-based segmentation, and FI volume was the most reliable metric overall. While FI cortical thickness had low overall intrasubject variability (low CoV), there was not much intersubject variability (low ICC). Data from the test–retest analysis area shown in Fig. 7A.

### Demographics

**Fig. 8:**
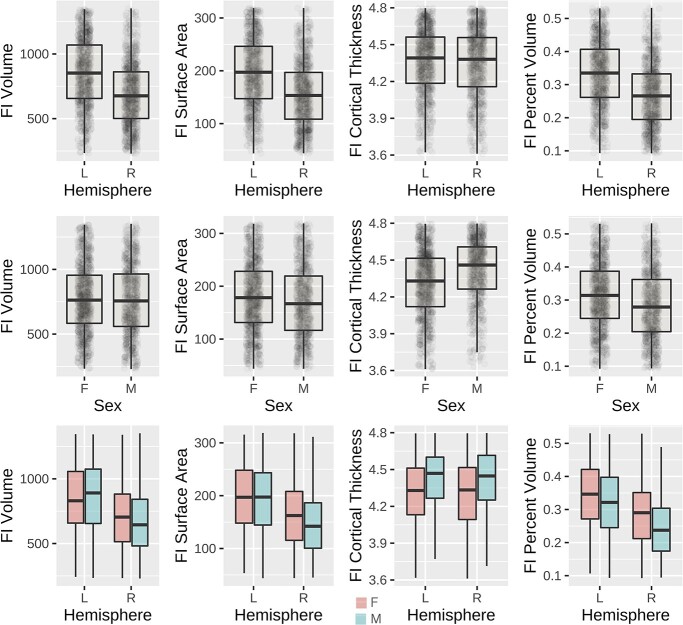
Plots showing differences in ODI-based FI volume between the left and right hemispheres (top row), between male and female participants (middle row), and further subdivisions (bottom row). An analogous plot for FA-based mapping can be found in the Supplementary Materials.

The results of our tests of lateralization and male–female differences are shown in Tables 2 and 3 and in Fig. 8. The findings for ODI-based segmentation were generally comparable to those obtained using FA-based segmentation with several exceptions that will be noted. Using ODI-based segmentation, these results indicate significant left lateralization of FI in volume and area, with a magnitude of 31% and 33%, respectively. We further found significantly greater FI surface area and percentage volume in females, with magnitudes of 7.4% and 15.3%. We found significantly greater FI thickness in males by 2.8%. The lateralization showed a strong effect, and the stronger male–female difference was in percentage FI volume. We also included covariates for whole-brain ODI and FA in these tests to account for any possible influence of whole-brain patterns on FI volume.

**Table 2:** Statistical differences between left and right hemispheres using ODI-based mapping. A negative value for Std. $\beta $ indicates that the measure is larger in the left hemisphere. Percentage measures were computed relative to each individuals total brain volume and area, respectively. An analogous table for FA-based mapping can be found in the Supplementary Materials.

Variable	R$^2$	Std. $\beta $	SE	$t$ -value	$P$ -value	$\Delta $ BIC
FI volume	0.223	–0.701	0.039	–18.147	$< 10^{-3}$	298.650
FI percent volume	0.217	–0.686	0.039	–17.700	$< 10^{-3}$	284.759
FI surface area	0.216	–0.702	0.039	–18.111	$< 10^{-3}$	297.561
FI percent area	0.229	–0.689	0.038	–17.934	$< 10^{-3}$	292.043
FI mean thickness	0.013	–0.033	0.044	–0.736	0.462	–7.068

**Table 3:** Statistical differences between male and female participants using ODI-based mapping. A negative value for Std. $\beta $ indicates that the measure is larger in females than males. Percentage measures were computed relative to each individuals total brain volume and area, respectively. An analogous table for FA-based mapping can be found in the Supplementary Materials.

Variable	R$^2$	Std. $\beta $	SE	$t$ -value	$P$ -value	$\Delta $ BIC
FI volume	0.163	–0.163	0.073	–2.218	0.027	–2.025
FI percent volume	0.149	–0.254	0.074	–3.434	0.001	4.822
FI surface area	0.125	–0.007	0.060	–0.123	0.902	–6.938
FI percent area	0.174	–0.282	0.073	–3.857	$< 10^{-3}$	7.875
FI mean thickness	0.073	0.635	0.077	8.197	$< 10^{-3}$	58.312

Our analysis of heritability using ODI-based segmentation showed that: FI volume had $r_{MZ} = 0.61$, $r_{DZ} =0.35$, $r_{NT} = 0.28$, and $H^{2}_{b} = 0.52$; FI surface area had $r_{MZ} = 0.60$, $r_{DZ} = 0.39$, $r_{NT} = 0.30$, and $H^{2}_{b} = 0.42$; FI thickness had $r_{MZ} = 0.24$, $r_{DZ} = 0.13$, $r_{NT} = 0.03$, and $H^{2}_{b} = 0.23$. Thus, FI volume and surface area had comparable heritability, whereas thickness was substantially lower. Data from the test–retest analysis area shown in Fig. 7B–D. For comparison, test–retest scans of the same individual had an FI volume correlation coefficient of 0.81.

### Behavioral variables

**Fig. 9:**
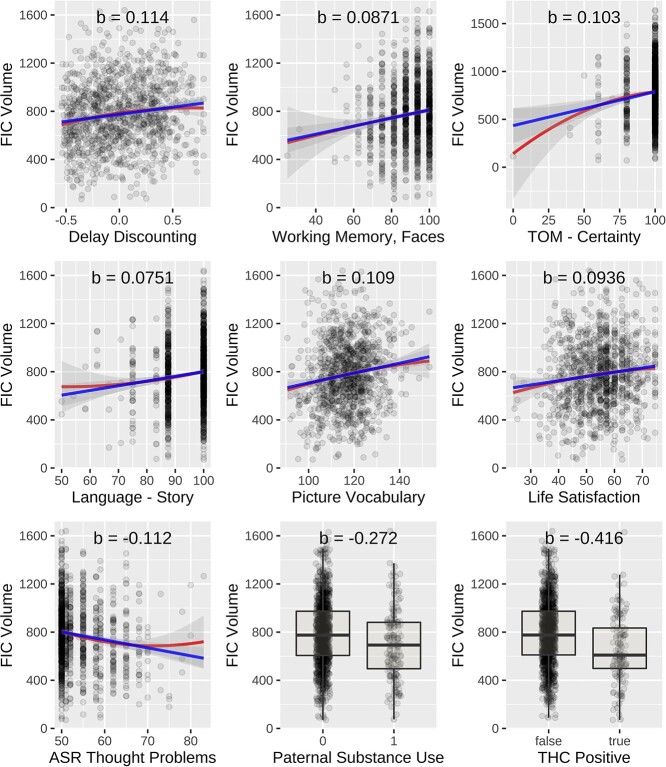
Plots showing statistically significant relationship between ODI-based FI volume and behavioral variables. The plots show FI volume computed from the average of the left and right hemispheres. Analogous plots for FA-based mapping can be found in the Supplementary Materials.

Our analysis of behavioral variables are summarized in Table 4 and Fig. 9. We found a total of 27 behavioral variables had a significant relationship with FI volume. Measures that were significant for both ODI- and FA-based delineation included: delay discounting (from 15 distinct combinations of time duration and monetary value), ASR thought problems, a history of paternal alcohol abuse, life satisfaction, picture vocabulary, TOM perception, TOM certainty, THC exposure, working memory task accuracy for faces. The language task accuracy for stories was only significant for ODI-based segmentation, and the following measures were significant using only FA-based segmentation: ER40 anger recognition, perceived hostility, and overall working memory accuracy. In testing thresholds that were above and below the optimum by 0.025, and we found that these results were more or less stable across those thresholds. A complete summary of these statistical results may be found in the Supplementary Material. Each of these variables is discussed in detail in the following section.

**Table 4:** Statistical results for behavioral variables using ODI-based mapping. An analogous table for FA-based mapping can be found in the Supplementary Materials.

Variable	R$^2$	Std. $\beta $	SE	$t$ -value	$P$ -value	$\Delta $ BIC
Delay discounting	0.162	0.114	0.029	3.973	$< 10^{-3}$	8.774
Working memory, faces	0.150	0.087	0.029	2.984	0.003	1.958
TOM, perception	0.159	0.086	0.029	2.965	0.003	1.855
TOM, certainty	0.163	0.103	0.029	3.584	$< 10^{-3}$	5.885
Language task, story accuracy	0.157	0.075	0.029	2.589	0.010	−0.227
Picture vocabulary	0.161	0.109	0.029	3.743	$< 10^{-3}$	7.021
Life satisfaction	0.158	0.094	0.029	3.274	0.001	3.757
ASR thought problems	0.162	–0.112	0.028	–3.951	$< 10^{-3}$	8.606
Paternal substance abuse	0.157	–0.272	0.084	–3.244	0.001	3.562
THC exposure	0.166	–0.416	0.092	–4.526	$< 10^{-3}$	13.407
Anger	0.153	0.066	0.029	2.307	0.021	–1.622
Perceived hostility	0.154	0.070	0.029	2.439	0.015	–0.997
Working memory accuracy	0.154	0.069	0.030	2.329	0.020	–1.520

## Discussion

Taken as a whole, the results of our experiments indicate that FI volume estimates using our approach are reliable, heritable, lateralized, sex dependent, and sensitive to behavioral measures of decision-making, emotion, and social behavior. As follows, we discuss the relation of our findings to previous work and highlight their implications.

### Relation to FI cytoarchitecture

Our primary finding is that FI can be distinguished from surrounding cortex by values below a threshold for the ODI, which implies a higher degree of membrane alignment channeling the directional movement of water molecules or by exceeding threshold values for FA, which similarly implies a higher degree of membrane alignment. Despite having different underlying modeling assumptions, the applications of these metrics yield converging delineations of FI. These results do not depend on the exact threshold since similar results were obtained with thresholds close to the optima. Furthermore, mapping of the principal diffusion direction in each FI voxel reveals that it is oriented perpendicular to the cortical surface (Fig. 6), which implies the presence of microstructural features in FI that favor this direction, FI is distinguished by large elongated bipolar neurons, which have been termed the VENs (Allman et al. 2010, Kim et al. 2012, Seeley et al. 2012, Von Economo 1926, Von Economo and Koskinas 1925). The elongated cell bodies of the VENs are located mainly in layer 5 with each VEN having a single large apical dendrite extending toward the cortical surface and a single large basal dendrite extending toward the white matter with adjacent VENs dendrites aligning parallel with each other and thus sharing a common spatial orientation perpendicular to the cortical surface (Allman et al. 2010, Kim et al. 2012, Watson et al. 2006). We further investigated the relationship between the VEN area and our resulting FI maps, and for this, we compared an a priori delineation of FI created for a previous study by co-author JMA based on his experience studying VENs at a cellular level in human tissue in serial whole-brain histological sections from the Yakovlev–Haleem Collection (Allman et al. 2010). Figure 4 shows substantial agreement between this a priori manually defined FI mask and those produced using the present algorithmic approach. We have also observed radially oriented arrays of axons in layers 5 and 6 in FI that might also contribute to the perpendicular direction. Another possible source might be the lancet-shaped pyramidal neurons described in FI by Rose (1928) that are distinct from the VENs.

### FI and self-control

Functional imaging and neurological data suggest that one of the basic functions of FI is self-control, particularly in social situations in the context of social rules and norms. FI is activated by the anticipation of risk (Mohr et al. 2010), and deficiency in risk anticipation may contribute to rule breaking behavior. The anterior insula including FI is also activated by the conscious awareness of having committed an error (Klein et al. 2013), and deficits in error recognition may also contribute to rule-breaking behavior. The successful suppression of impulsivity in the GO-NOGO task is linked to strong activation of FI (Dambacher et al. 2015). FI is also one of the sites of degeneration in the bvFTD (Kim et al. 2012), which is characterized by loss of self-control and social norm violation (Lough et al. 2006). In a large study of patients who had been diagnosed with bvFTD with high confidence, nearly all had bilateral atrophy of FI (Perry  et al. 2017). In subjects who are carriers for genetic mutations that cause bvFTD, behavioral disinhibition is one of the earliest symptoms to become manifest (Benussi et al. 2021). The loss of VENs in the right FI in the early stages of symptomatic bvFTD is strongly associated with disinhibition (Kim et al. 2012).

Delay discounting is the preference for immediate rewards versus those that require a waiting period as measured by the reduced size of the immediate reward accepted compared with the delayed reward offered. The volume of FI is linked to many measures of delay discounting with larger discounting related to smaller FI size (Table 4, Fig. 9). Numerous studies indicate that greater delay discounting is related to poorer self-control (Sadeh and Clopath 2021). Delay discounting has been specifically linked to tobacco, alcohol, and cannabis use in the HCP dataset (Naudé et al. 2021). A recent carefully controlled study of cannabis users found both increased delay discounting and impulsivity (O’Donnell et al. 2021). FI volume is also reduced in subjects who had a father with either drug or alcohol problems (paternal substance abuse). Our analysis also supports previous findings of FI changes associated with THC exposure (Cabeen et al. 2020), but with a larger sample size and specifically with FI volume. The THC finding is distinctive among our tested measures, as it was obtained from biospecimen tests without the usual caveats related to self-reported substance use.

Another measure of self-control is the ability to suppress the outward expression of emotions (expressive suppression), which is positively correlated with anterior insula volume in normal women (Giuliani et al. 2011); only women were tested in the cited work. The ability to suppress the outward expression of emotions is particularly weak in subjects with bvFTD, and the volume of the insula is commensurately smaller in them than in healthy controls (Muhtadie et al. 2021).

FI volume is reduced in individuals who have difficulty suppressing intrusive or self-destructive thoughts, as revealed by higher scores in the measure termed thought problems or negative intrusive thinking (Table 4, Fig. 9). This finding implies that difficulties with self-control extend to the regulation of the thinking process itself. This result is consistent with our earlier findings for FI of reduced FA and increased ODI for subjects with intrusive thinking (Cabeen et al. 2020).

### FI and social functioning

Subjects with bvFTD have difficulties interpreting spoken sarcastic statements in which the true meaning is the opposite of the literal interpretation (Kipps et al. 2009). These difficulties understanding sarcastic speech has been attributed to a reduced capacity to discriminate negative emotions (Kipps et al. 2009), but it may also arise from a deficit in detecting the contradictions inherent in the sense of humor. Reduced sense of humor is more profound in bvFTD than in other dementias (Clark et al. 2016). FI is activated in proportion to the degree of funniness of cartoons (Watson et al. 2007) and the connection between FI and humor has recently been confirmed by a large meta-analysis of many more recent imaging studies (Farkas et al. 2021). 

The connection between social functioning and FI is further supported by pathological changes in the location of the expression of the protein TDP-43 in the VENs and closely related Fork cells in FI in the right hemisphere in bvFTD (Pasquini et al. 2020). In VENs and Fork cells that are not undergoing degeneration, the expression of TDP-43 is restricted to the nucleus, but in those cells that have begun to degenerate the expression of TDP-43 migrates to the cytoplasm, and ultimately the nuclear expression of TDP-43 is depleted (Pasquini et al. 2020). This translocation and depletion of TDP-43 expression is correlated with degenerative changes in the connections of FI and the loss of emotional empathy in these patients (Pasquini et al. 2020).

FI’s role in social functioning is also implicated in another form of FTD, the semantic variant of primary progressive aphasia (svPPA) that directly affects the anterior temporal lobe with secondary affects elsewhere in the brain. In svPPA, the connections between the anterior temporal lobe and FI are reduced compared with normal subjects and the responses to facial expressions are reduced across a broad range of emotions (Guo et al. 2013). Working memory for faces is positively related to FI volume in our analysis.

The relationship between FI and more abstract aspects of social functioning is revealed by the increased FI volume with better performance on a task designed to measure the capacity for TOM. Accurate insights concerning the mental states of others may serve as an important restraint on impulsivity and favor self-control in social interactions. The capacity for theory of mind is deficient in bvFTD when subjects are compared either to normal individuals or to people with Alzheimer’s disease (Bora et al. 2015).

Consistent with our earlier findings (Cabeen et al. 2021), we found that the FI volume increases as a function of life satisfaction bilaterally and in the right hemisphere considered separately. The measure of life satisfaction is related to Easterlin’s “happiness,” which is an aggregation of satisfaction with employment, friends and family relationships, and health (Easterlin, 2021). The HCP data suggest that these affect the microstructure and volume of FI.

### Differences in FI cortical volume fraction in men and women

In FI, the fraction of the total volume of cerebral cortex occupied by FI is greater in women than in men (Table 3, Fig. 8). Based on the HCP data, responses to both reward and loss in the gambling task elicited greater activity in women than men for brain coordinates centered on FI (Li et al. 2020). In a direct measure of self-control, women responding in the STOP signal task had greater activity than men for the brain coordinates centered on FI in the left hemisphere (Gaillard et al. 2020). When women gazed on pictures of their own children versus other children whom they knew, FI in the left hemisphere was activated (Leibenluft et al. 2004). FI is also activated when women responded to the cries of their own infant versus another’s infant (Lorberbaum et al. 2002, Riem et al. 2011, Swain et al. 2008). FI activity was also related in women to emotional closeness with their partners (Ortigue et al. 2007). These findings with respect to reward, punishment, self-control, love, parenting, and partner bonding suggest possible reasons why FI occupies a greater volume fraction in women than in men. FI is strongly implicated in bvFTD (Kim et al. 2012, Pasquini et al. 2020, Perry et al. 2017), and the larger FI volume fraction might explain why women with bvFTD possess greater reserve with respect to many prominent symptoms including executive functioning, apathy, appetite regulation, and sleep disturbances and that neurodegeneration must be more severe in women to produce symptoms of equivalent severity to men (Illán-Gala et al. 2021). Given that FI has a role in the regulation of impulsivity and autonomic responses (Allman et al. 2010, Guo et al. 2016, Kim et al. 2012, Mutschler et al. 2009), it’s larger cortical volume fraction in women may be part of the neuronal circuity responsible for the lower risks in women of dying from violence, accidents, ischemic heart disease, and stroke throughout life (Allman and Hasenstaub 2001).

### FI volume: hemispheric differences, language, and cognition

FI volume is greater in the left hemisphere than the right (Table 2, Fig. 8). An earlier study of volumetric measurements in FI based directly on serial Nissl-stained histological sections in 5 individuals found that FI was strongly skewed to the left side in 3 of 5 subjects with the other 2 slightly skewed to the right (Bauernfeind et al. 2013). The leftward asymmetry in FI volume may be related to linguistic functions in FI. The picture vocabulary task in which subjects match pictures of objects to spoken words and other measures of language function such as language task accuracy are positively related to FI volume in the left hemisphere (Table 4, Fig. 9). These language-related functions in FI may be a possible explanation for its larger volume in the left hemisphere, which would be consistent with left hemisphere specialization for language functions (Sperry 1982). Several measures of working memory are positively related to FI volume bilaterally and in the right hemisphere considered separately. Working memory is required for the neural computation of the risk and pain prediction error signals observed in FI in functional imaging experiments (Fazeli and Büchel 2018, Preuschoff et al. 2008). Working memory contributes the capacity to anticipate risk. Working memory also contributes to many aspects of social functioning such as TOM.

The VENs are more numerous in the right FI than the left (Allman et al. 2010), which is consistent with their role in regulation of impulsivity and self-control in the right FI (Kim et al. 2012). However as noted above, FI volume is greater in the left FI than the right (Table 2, Fig. 8). Other elongated structures in FI-orientated perpendicular to the cortical surface, such as the axon arrays in layers 5–6 and the lancet-shaped neurons, might give rise to measures of membrane alignment. In his comprehensive study of the cytoarchitecture of the insular cortex, Rose (1928) found mixed with the VENs in layer 5 of FI, a population of elongated lancet-shaped pyramidal neurons with their apical dendrites extending parallel to the apical dendrites of the VENs but having multiple basal dendrites unlike the single basal dendrite in the VENs. There has been no quantification of these lancet-shaped pyramidal neurons, but these neurons or other factors could be related to the leftward skew in FI volume revealed by the ODI-FA mapping.

### Variance and limitations of FI mapping

Considering the R$^2$ values (Table 4) or the percentage of variance attributable to each correlation, the strongest effects are hemispheric differences favoring FI in the left versus the right hemisphere, which are around 12%. In the biological variables, the variance associated with greater FI thickness in males versus females is 6.7%. However, this strongly contrasts with greater FI surface area and cortical volume fraction, which favors women with variances of 6.1% and 4.68%, respectively. The various significant behavioral parameters have R$^2$ values of around 2–3%. Data from MZ and DZ and unrelated subjects indicate that there is a moderate degree of heritability of FI volume and surface area, which is in line with previous heritability estimates of brain structure (Gilmore et al. 2010, Peper et al. 2007, Zhao et al. 2019). Another important caveat is that we found the whole-brain average of cortical ODI explained some variance (13%), and because age-related changes have been reported in whole neocortical ODI (Nazeri et al. 2015, Vogt et al. 2020), this could introduce a bias that would require care and further consideration to handle. This suggests there is some genetic component to the development of FI, but in additional other factors yet to be discovered may play a role in influencing the size of FI, for example factors related to environmental and measurement error during scanning. Finally, it will be important to validate this work at a cellular level to understand precisely which cytoarchitectural features contribute to changes in diffusion microstructure, and this may be investigated through a combined analysis of post-mortem tissue with high-resolution MRI and microscopy of the same specimen.

### Microstructure and homeostatic mechanisms

The following observations link FI and the VENs to homeostatic mechanisms. In mice, FEZF2-positive neurons in the anterior insula that project to the brainstem are directly related to homeostatic responses to challenges such as water deprivation, but not to overall consumption (Deng et al. 2021). In humans, FI is activated by the conscious recognition of error, and pain and risk prediction error signals have been recorded from FI, which could trigger targeted homeostatic responses (Fazeli and Büchel 2018, Klein et al. 2013, Preuschoff et al. 2008). Thus these neurons are related to targeted responses to homeostatic challenges and not to general appetite.In the anterior insula in primates, the FEZF2-positive neurons also project to the brain stem (Cobos and Seeley 2015, Evrard 2019, Hodge et al. 2020). In both mice and primates, they project to the parabrachial nucleus (Deng et al. 2021, Evrard 2019), which is crucially involved in homeostatic mechanisms (Chiang et al. 2019). In FI, the VENs express FEZF2 (Cobos and Seeley, 2015, Hodge et al. 2020). Thus, while there do not appear to be neurons with the VEN morphology in the anterior insula in mice, there are layer 5 neurons that share anterior insular location, FEZF2 expression and projection to the brain stem. This relationship between the FI-VENs and the layer 5 anterior insula neurons in mice is further supported by the expression of CTIP2 in neurons projecting to the parabrachial nucleus in mice (Grady et al. 2020) since CTIP2 is also selectively expressed in the FI-VENs in humans (Cobos and Seeley, 2015). The implication is that this pathway is related to responses to homeostatic challenges in mice and primates and perhaps in mammals generally. In some highly social species, these homeostatic challenges may extend to sustaining the well-being of others and hence could be the basis for empathy, which is reduced in TDP-43 mediated pathology in the FI-VENs in the bvFTD (Pasquini et al. 2020). Livneh and Andermann (2021) have emphasized the role of insular circuitry in maintaining well-being and homeostatic regulation over different time scales, which is consistent with our finding that FI volume is related to life satisfaction. The axons and dendrites of the FEZF2-positive neurons may influence the diffusion signals used in our study to map FI. We have also found that FI volume is reduced in cannabis users. This observation extends our earlier findings (Cabeen et al. 2020), which we suggested might be related to the effects of THC exposure on presynaptic cannabinoid receptors in cholecystokinin basket cells mediated by the degradation of stathmin-2, a protein involved in axon growth and maintenance (Klim et al. 2019, Tortoriello et al. 2014).

## Conclusions

We developed a novel method for mapping the location, surface area, thickness, and volume of FI using a technique for delineating FI by jointly modeling cortical surface geometry and its coincident diffusion microstructure parameters. We found that diffusion MRI microstructure parameters (ODI and FA) in cortical gray matter can be used to map FI in specific individuals, and the derived measures reflect a range of behavioral factors in young adults from the HCP (*N*=1052). We found FI volume is associated with measures of decision making (delay discounting, substance abuse), emotion (negative intrusive thinking and perceived hostility), and social behavior (TOM and working memory for faces). The common denominator of these findings is that larger FI size is related to greater self-control and social awareness. We further found FI volume was substantially larger in the left hemisphere than the right (31%). We also found the percentage volume of FI compared with the total cortical volume is larger in women than men (15.3%). Since the study population contained substantial numbers of MZ and DZ and repeated scanning, we were able to demonstrate of moderate degree of heritability and reliability of FI volume. Our method reproduces previously reported results obtained with an ROI approach based on atlas registration (THC exposure, life satisfaction, negative intrusive thinking), but with higher sensitivity. Microstructure-based mapping of FI provides a novel measure that may reflect gray matter tissue cytoarchitecture with possible applications to understanding structural changes in FI across the lifespan and in neurodegeneration.

## Supplementary Material

FIC-Map-Supplement_bhac237

## Data Availability

Data used in our study is available with permission from the Human Connectome Project ^1^. Our data image analysis and visualization tools available online as part of the Quantitative Imaging Toolkit (QIT) ^2^,^3^.
