# Supplementary material for: Mapping frontoinsular cortex from diffusion microstructure
Source: Cereb Cortex. 2022 Jun 27;33(6):2715–33. doi: 10.1093/cercor/bhac237 (PMC10016069; doi:10.1093/cercor/bhac237)
Supplement: FIC-Map-Supplement_bhac237 [file fic-map-supplement_bhac237.pdf]

Supplementary materials for ‘Mapping frontoinsular cortex from diffusion microstructure’

Ryan P. Cabeen, Arthur W. Toga, John M. Allman

19 May, 2022

Included below are data visualizations, plots, tables that accompany the main manuscript of the paper *“Mapping frontoinsular cortex with diffusion microstructure”*. This includes a plot of the restriction surface mask, visualizations of the threshold optimization procedure, visualizations of example results, visualizations of population averaged results, tables reporting distributional parameters, tables reporting statistical effects with demographic variables, lateralization, differences between men and women, and behavioral variables. These figures were created for both ODI-based and FA-based FI mapping. For comparison, results are also included from a T1 MRI analysis of anterior agranular insular cortex (AAIC) using Freesurfer.

QIT Modules for FI Mapping

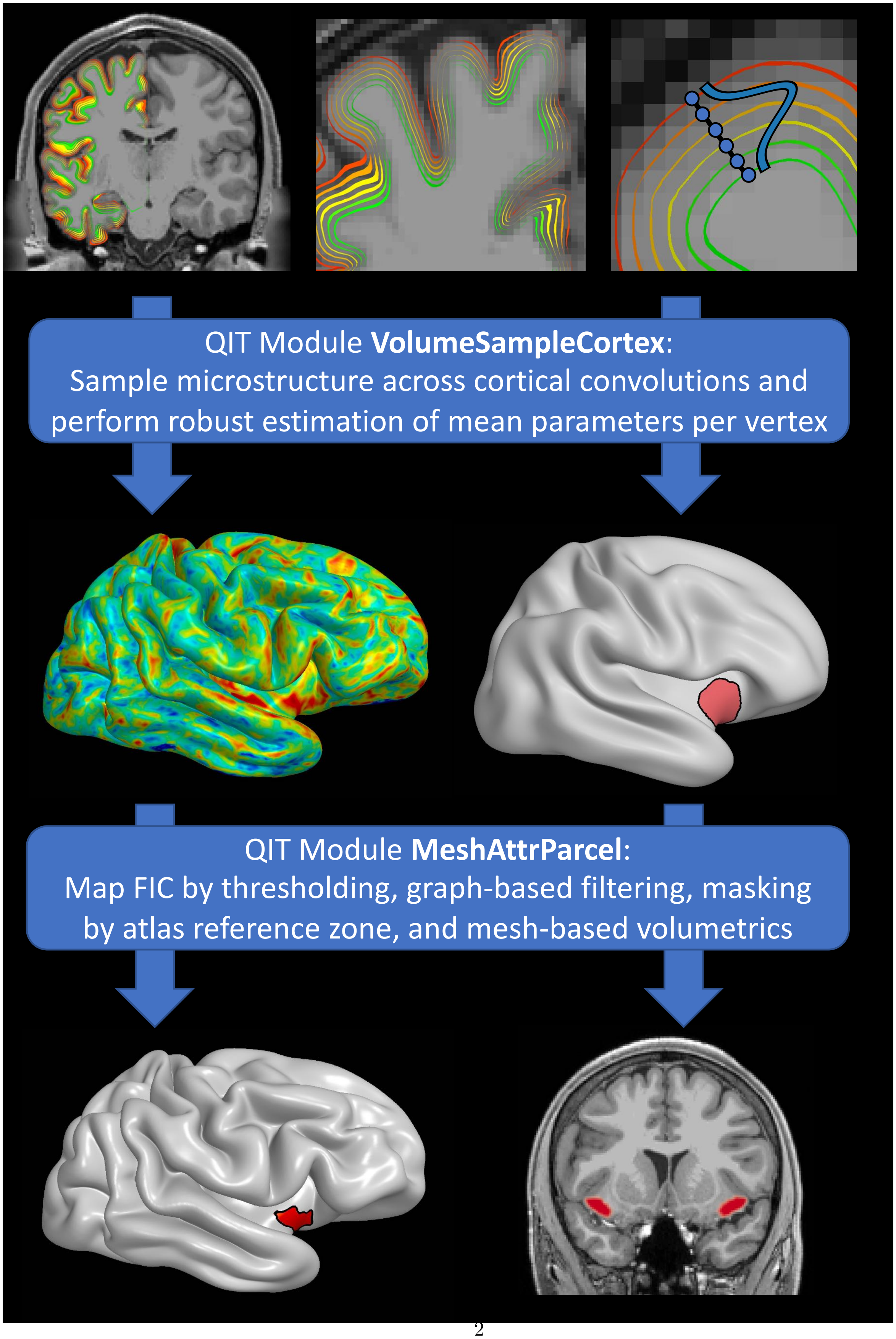

Threshold Optimization for ODI-based FI Mapping

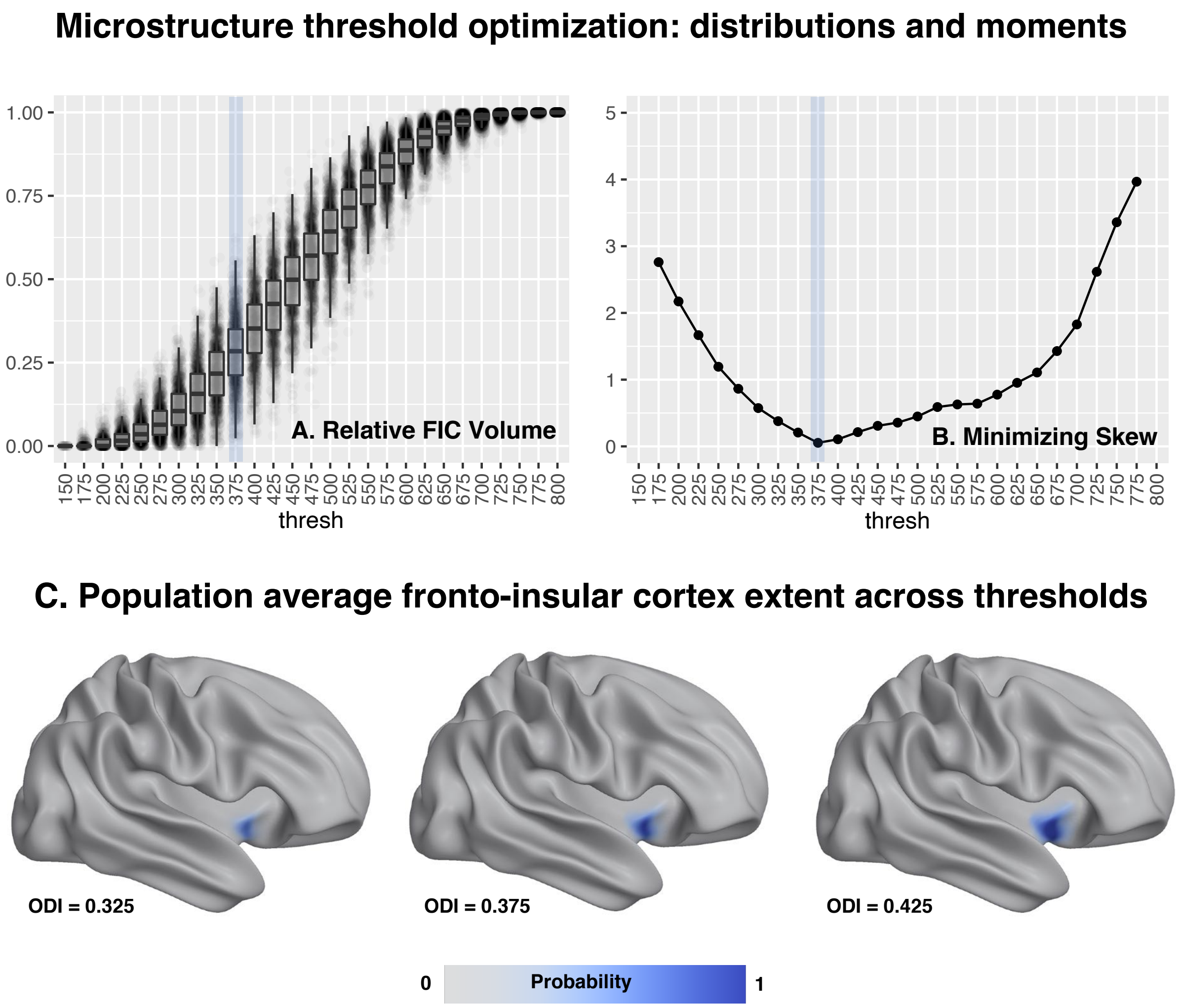

Threshold Optimization for FA-based FI Mapping

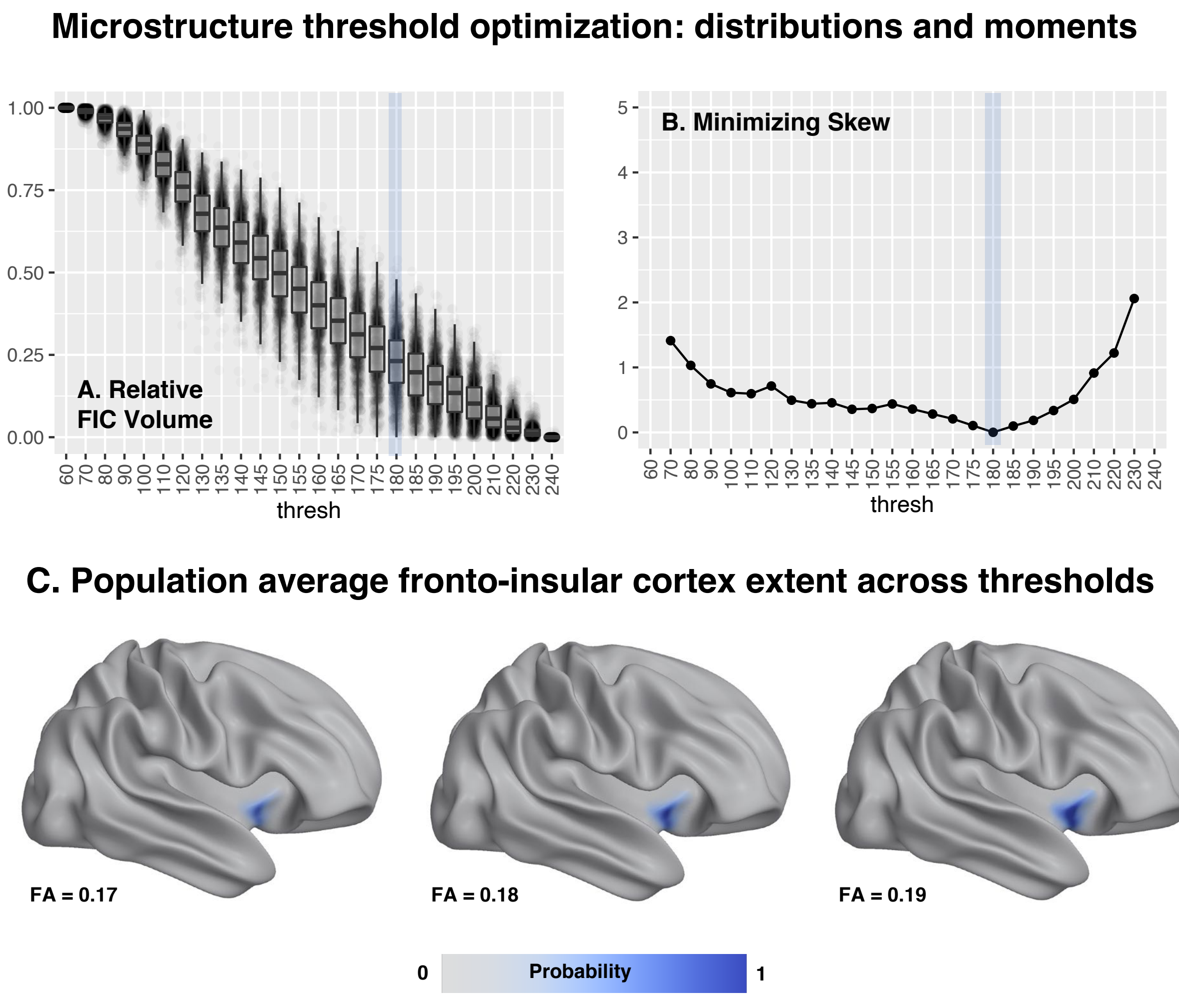

Example of Individual Results from ODI-based FI Mapping

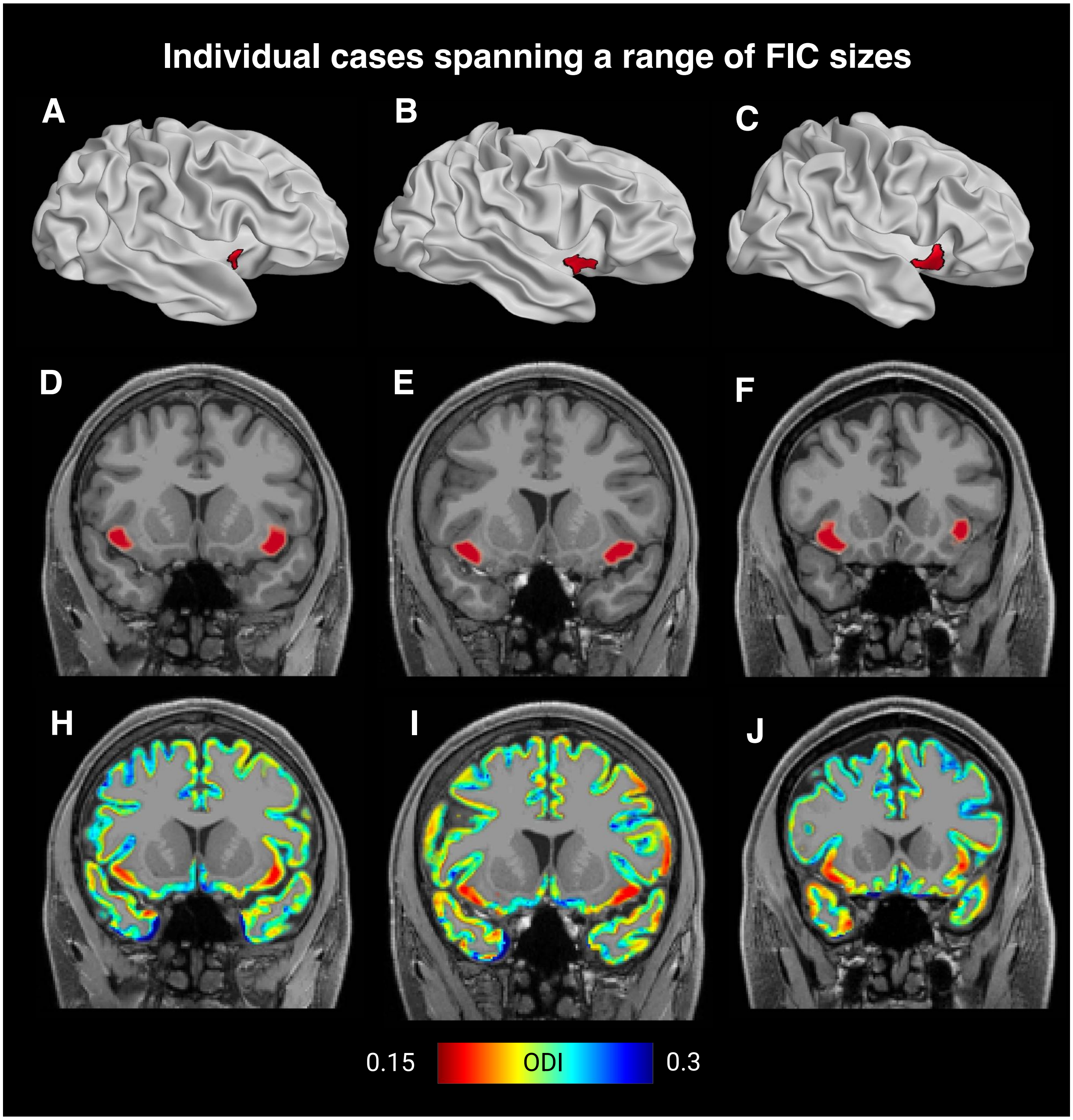

Example of Individual Results from FA-based FI Mapping

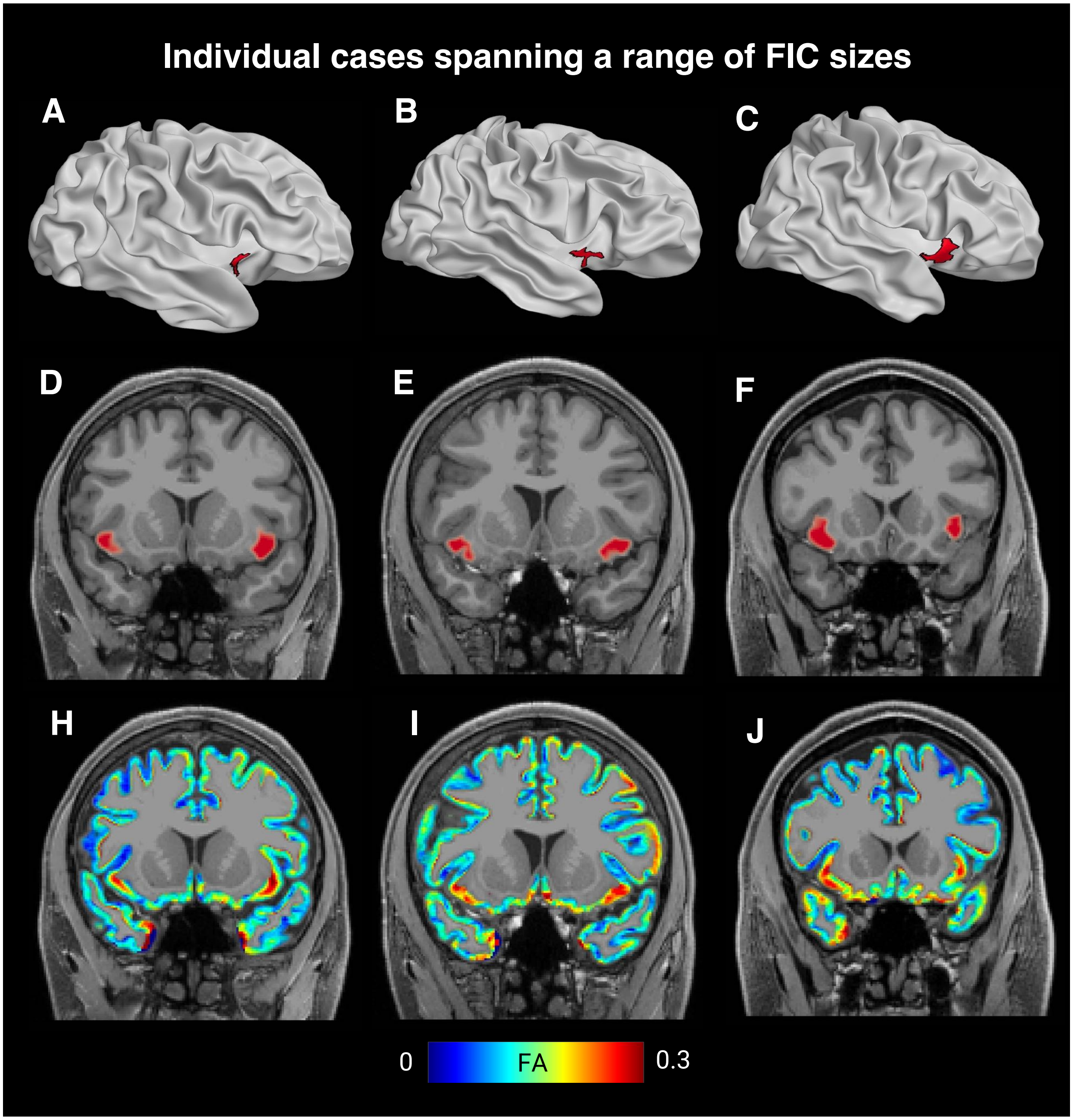

Population Average Results from ODI-based FI Mapping

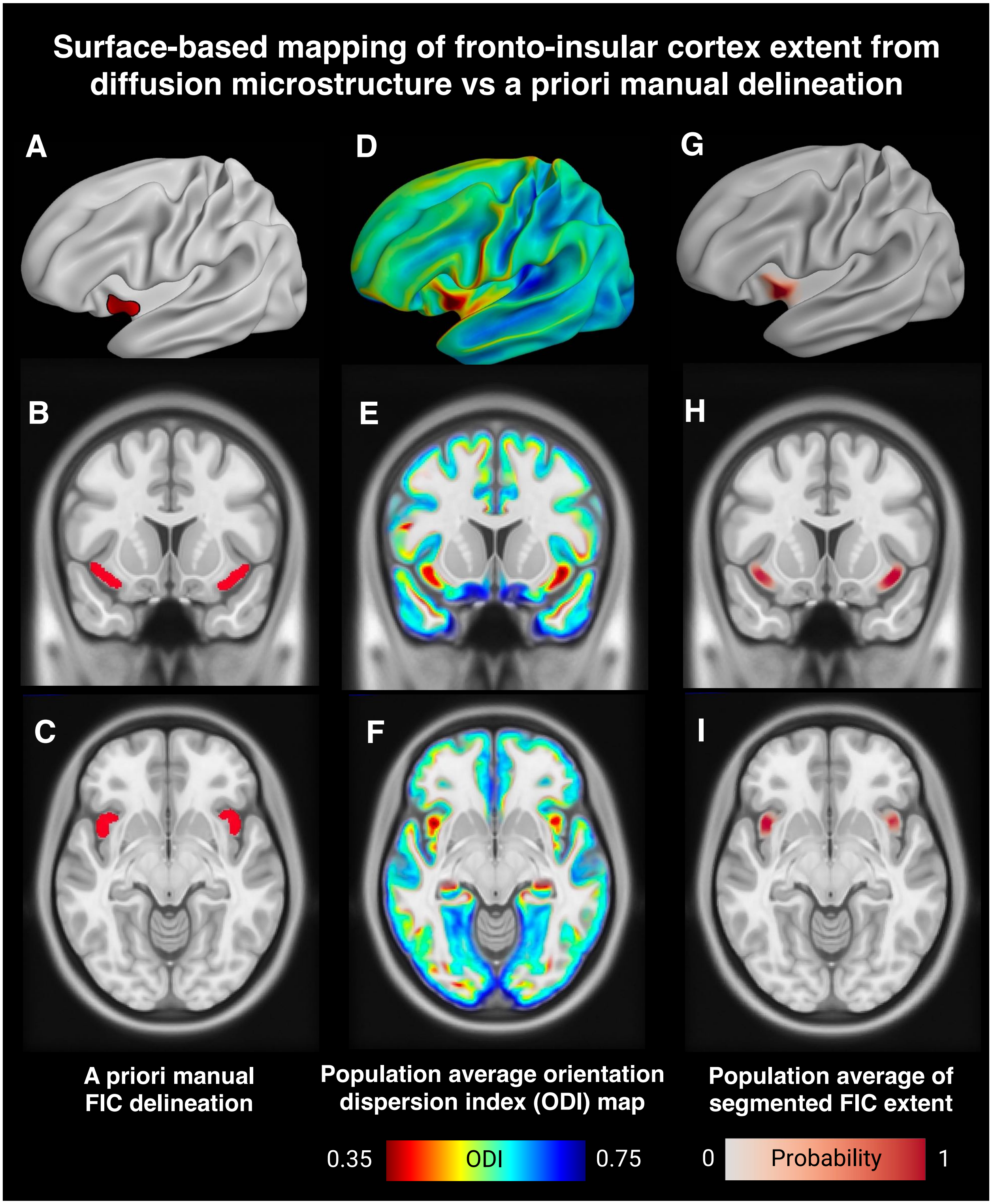

Population Average Results from FA-based FI Mapping

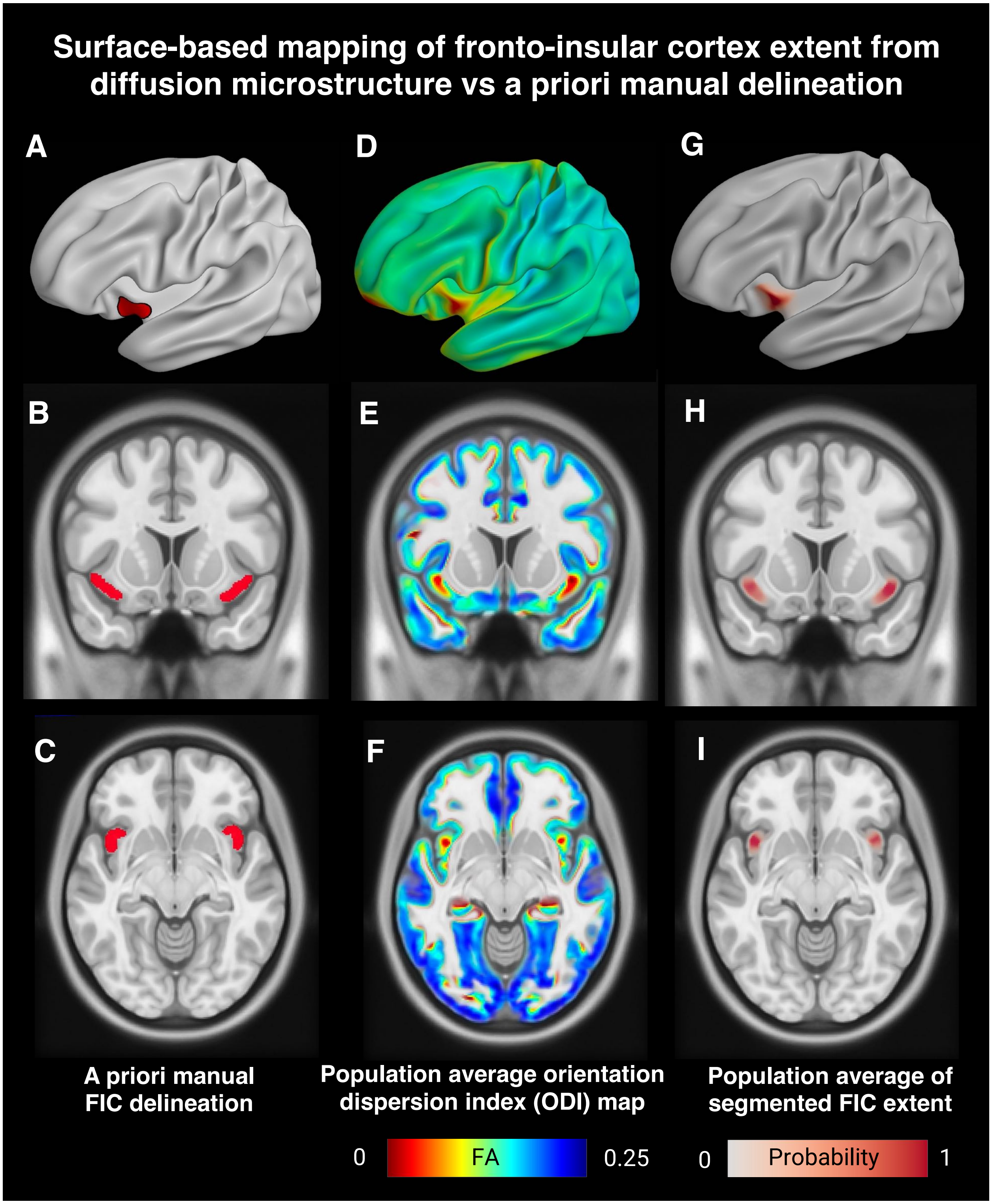

Distributional Summary from ODI-based FI Mapping

Table 1: A numerical summary of ODI-derived imaging metrics

	Mean	SD
FI Volume	776.469	277.297
FI Percent Volume	0.302	0.107
FI Surface Area	177.395	67.228
FI Percent Surface Area	0.189	0.073
FI Mean Thickness	4.304	0.341
Whole Cortex Volume	257738.432	27191.217
Whole Cortex Area	94711.004	9541.832
Whole Cortex Thickness	2.796	0.075
Whole Cortex ODI	0.579	0.012

Table 2: A numerical summary of ODI-derived imaging metrics split by hemisphere

	Hemisphere	Mean	SD
FI Volume	Left	897.742	337.026
	Right	654.632	300.216
FI Percent Volume	Left	0.349	0.128
	Right	0.255	0.117
FI Surface Area	Left	206.852	82.069
	Right	147.806	73.064
FI Percent Surface Area	Left	0.220	0.087
	Right	0.157	0.079
FI Mean Thickness	Left	4.355	0.320
	Right	4.252	0.542
Whole Cortex Volume	Left	257935.473	27277.641
	Right	257534.637	27153.484
Whole Cortex Area	Left	94591.239	9516.890
	Right	94828.810	9591.365
Whole Cortex Thickness	Left	2.800	0.076
	Right	2.791	0.075
Whole Cortex ODI	Left	0.576	0.013
	Right	0.581	0.013

Table 3: A numerical summary of ODI-derived imaging metrics split by sex

	Sex	Mean	SD
Age	Female	29.462	3.568
	Male	27.909	3.606
FI Volume	Female	785.204	270.127
	Male	766.118	285.497
FI Percent Volume	Female	0.323	0.107
	Male	0.277	0.101
FI Surface Area	Female	183.096	65.998
	Male	170.640	68.110
FI Percent Surface Area	Female	0.205	0.073
	Male	0.169	0.068
FI Mean Thickness	Female	4.249	0.327
	Male	4.369	0.345
Whole Cortex Volume	Female	242260.734	20163.589
	Male	276079.986	22667.373
Whole Cortex Area	Female	89340.165	7244.187
	Male	101075.616	7880.899
Whole Cortex Thickness	Female	2.789	0.069
	Male	2.805	0.081
Whole Cortex ODI	Female	0.576	0.012
	Male	0.582	0.012

Distributional Summary from FA-based FI Mapping

Table 4: A numerical summary of FA-derived imaging metrics

	Mean	SD
FI Volume	652.274	283.421
FI Percent Volume	0.253	0.106
FI Surface Area	149.506	67.961
FI Percent Surface Area	0.158	0.071
FI Mean Thickness	4.150	0.421
Whole Cortex Volume	257738.432	27191.217
Whole Cortex Area	94711.004	9541.832
Whole Cortex Thickness	2.796	0.075
Whole Cortex FA	0.114	0.004

Table 5: A numerical summary of FA-derived imaging metrics split by hemisphere

	Hemisphere	Mean	SD
FI Volume	Left	719.496	343.830
	Right	584.554	323.245
FI Percent Volume	Left	0.279	0.129
	Right	0.226	0.122
FI Surface Area	Left	164.787	82.774
	Right	134.111	77.339
FI Percent Surface Area	Left	0.174	0.087
	Right	0.142	0.081
FI Mean Thickness	Left	4.289	0.381
	Right	4.011	0.689
Whole Cortex Volume	Left	257935.473	27277.641
	Right	257534.637	27153.484
Whole Cortex Area	Left	94591.239	9516.890
	Right	94828.810	9591.365
Whole Cortex Thickness	Left	2.800	0.076
	Right	2.791	0.075
Whole Cortex FA	Left	0.114	0.005
	Right	0.114	0.005

Table 6: A numerical summary of FA-derived imaging metrics split by sex

	Sex	Mean	SD
Age	Female	29.462	3.568
	Male	27.909	3.606
FI Volume	Female	635.165	269.847
	Male	672.548	297.720
FI Percent Volume	Female	0.261	0.107
	Male	0.243	0.104
FI Surface Area	Female	148.503	66.053
	Male	150.696	70.206
FI Percent Surface Area	Female	0.166	0.072
	Male	0.149	0.069
FI Mean Thickness	Female	4.113	0.415
	Male	4.195	0.424
Whole Cortex Volume	Female	242260.734	20163.589
	Male	276079.986	22667.373
Whole Cortex Area	Female	89340.165	7244.187
	Male	101075.616	7880.899
Whole Cortex Thickness	Female	2.789	0.069
	Male	2.805	0.081
Whole Cortex FA	Female	0.115	0.004
	Male	0.113	0.004

Effects of Demographic Variables from ODI-based FI Mapping

Table 7: Sex differences using ODI segmentation. Negative beta values indicate that the parameter was larger in female participants.

Name	RSq	Beta	StdErr	tValue	pValue	dBIC
FI Volume	0.163	-0.163	0.073	-2.218	0.027	-2.025
FI Percent Volume	0.149	-0.254	0.074	-3.434	0.001	4.822
FI Surface Area	0.125	-0.007	0.060	-0.123	0.902	-6.938
FI Percent Area	0.174	-0.282	0.073	-3.857	0.000	7.875
FI Mean Thickness	0.073	0.635	0.077	8.197	0.000	58.312
Whole Cortex Thickness	0.016	0.252	0.062	4.079	0.000	9.593
Whole Cortex Area	0.376	1.230	0.049	25.060	0.000	485.687
Whole Cortex Volume	0.384	1.244	0.049	25.508	0.000	499.645
Whole Cortex ODI	0.053	0.463	0.061	7.627	0.000	49.761

Table 8: Hemispheric differences using ODI-based segmentation. Negative beta values indicate the parameter is larger in the left hemisphere than the right.

Name	RSq	Beta	StdErr	tValue	pValue	dBIC
FI Volume	0.223	-0.701	0.039	-18.147	0.000	298.650
FI Percent Volume	0.217	-0.686	0.039	-17.700	0.000	284.759
FI Surface Area	0.216	-0.702	0.039	-18.111	0.000	297.561
FI Percent Area	0.229	-0.689	0.038	-17.934	0.000	292.043
FI Mean Thickness	0.013	-0.033	0.044	-0.736	0.462	-7.068
Whole Cortex Thickness	0.003	-0.114	0.044	-2.596	0.010	-0.909
Whole Cortex Area	0.000	0.025	0.044	0.579	0.563	-7.311
Whole Cortex Volume	0.000	-0.015	0.044	-0.339	0.734	-7.530
Whole Cortex ODI	0.035	0.376	0.043	8.749	0.000	67.596

Effects of Demographic Variables from FA-based FI Mapping

Table 9: Sex differences using FA-based segmentation. Negative beta values indicate that the parameter was larger in female participants.

Name	RSq	Beta	StdErr	tValue	pValue	dBIC
FI Volume	0.134	-0.105	0.075	-1.400	0.162	-4.984
FI Percent Volume	0.079	-0.054	0.062	-0.868	0.386	-6.192
FI Surface Area	0.126	-0.182	0.075	-2.423	0.016	-1.072
FI Percent Area	0.092	-0.093	0.061	-1.525	0.128	-4.619
FI Mean Thickness	0.024	0.270	0.065	4.172	0.000	10.400
Whole Cortex Thickness	0.016	0.252	0.062	4.079	0.000	9.593
Whole Cortex Area	0.376	1.230	0.049	25.060	0.000	485.687
Whole Cortex Volume	0.384	1.244	0.049	25.508	0.000	499.645
Whole Cortex ODI	0.004	0.123	0.044	2.792	0.005	0.150

Table 10: Hemispheric differences using FA-based segmentation. Negative beta values indicate the parameter is larger in the left hemisphere than the right.

Name	RSq	Beta	StdErr	tValue	pValue	dBIC
FI Volume	0.127	-0.380	0.041	-9.289	0.000	77.104
FI Percent Volume	0.091	-0.378	0.042	-9.052	0.000	72.875
FI Surface Area	0.119	-0.363	0.041	-8.838	0.000	69.218
FI Percent Area	0.102	-0.376	0.041	-9.070	0.000	73.194
FI Mean Thickness	0.026	-0.259	0.045	-5.777	0.000	25.564
Whole Cortex Thickness	0.003	-0.114	0.044	-2.596	0.010	-0.909
Whole Cortex Area	0.000	0.025	0.044	0.579	0.563	-7.311
Whole Cortex Volume	0.000	-0.015	0.044	-0.339	0.734	-7.530
Whole Cortex ODI	0.048	0.436	0.043	10.171	0.000	93.410

Statistics of Behavioral Variables from ODI-based FI Mapping

Table 11: Statistical results using ODI-based segmentation. Positive beta scores indicate that increasing parameter values correspond to a larger FI volume.

Variable	RSq	Beta	StdErr	tValue	pValue	dBIC
Delay Discounting	0.162	0.114	0.029	3.973	0.000	8.774
Working Memory, Faces	0.150	0.087	0.029	2.984	0.003	1.958
Theory of Mind, Perception	0.159	0.086	0.029	2.965	0.003	1.855
Theory of Mind, Certainty	0.163	0.103	0.029	3.584	0.000	5.885
Language Task, Story Accuracy	0.157	0.075	0.029	2.589	0.010	-0.227
Picture Vocabulary	0.161	0.109	0.029	3.743	0.000	7.021
Life Satisfaction	0.158	0.094	0.029	3.274	0.001	3.757
ASR Thought Problems	0.162	-0.112	0.028	-3.951	0.000	8.606
Paternal Substance Abuse	0.157	-0.272	0.084	-3.244	0.001	3.562
THC Exposure	0.166	-0.416	0.092	-4.526	0.000	13.407
Anger	0.153	0.066	0.029	2.307	0.021	-1.622
Perceived Hostility	0.154	0.070	0.029	2.439	0.015	-0.997
Working Memory Accuracy	0.154	0.069	0.030	2.329	0.020	-1.520

Statistics of Behavioral Variables from FA-based FI Mapping

Table 12: Statistical results using FA-based segmentation. Positive beta scores indicate that increasing parameter values correspond to a larger FI volume.

Variable	RSq	Beta	StdErr	tValue	pValue	dBIC
Delay Discounting	0.129	0.125	0.029	4.285	0.000	11.333
Working Memory, Faces	0.117	0.103	0.030	3.449	0.001	4.941
Theory of Mind, Perception	0.128	0.105	0.030	3.543	0.000	5.608
Theory of Mind, Certainty	0.129	0.108	0.029	3.667	0.000	6.495
Language Task, Story Accuracy	0.123	0.073	0.030	2.460	0.014	-0.870
Picture Vocabulary	0.130	0.131	0.030	4.375	0.000	12.102
Life Satisfaction	0.122	0.088	0.029	2.985	0.003	1.963
ASR Thought Problems	0.123	-0.094	0.029	-3.229	0.001	3.475
Paternal Substance Abuse	0.125	-0.303	0.085	-3.543	0.000	5.589
THC Exposure	0.127	-0.363	0.094	-3.858	0.000	7.901
Anger	0.121	0.088	0.029	3.019	0.003	2.169
Perceived Hostility	0.121	0.079	0.029	2.699	0.007	0.343
Working Memory Accuracy	0.121	0.084	0.031	2.752	0.006	0.629

Statistics of Behavioral Variables from AAIC Region Volume

Table 13: Statistical results using Freesurfer-based AAIC segmentation. Positive beta scores indicate that increasing parameter values correspond to a larger FI volume.

Variable	RSq	Beta	StdErr	tValue	pValue	dBIC
Delay Discounting	0.039	0.053	0.031	1.726	0.085	-3.957
Working Memory, Faces	0.038	0.059	0.031	1.903	0.057	-3.302
Theory of Mind, Perception	0.037	-0.001	0.031	-0.027	0.979	-6.926
Theory of Mind, Certainty	0.038	0.034	0.031	1.085	0.278	-5.744
Language Task, Story Accuracy	0.039	0.036	0.031	1.162	0.246	-5.572
Picture Vocabulary	0.040	0.067	0.031	2.155	0.031	-2.291
Life Satisfaction	0.039	0.060	0.031	1.960	0.050	-3.093
ASR Thought Problems	0.036	-0.041	0.031	-1.326	0.185	-5.178
Paternal Substance Abuse	0.039	-0.180	0.091	-1.980	0.048	-3.010
THC Exposure	0.039	-0.178	0.100	-1.780	0.075	-3.767
Anger	0.036	0.013	0.031	0.424	0.672	-6.763
Perceived Hostility	0.037	0.031	0.031	0.995	0.320	-5.951
Working Memory Accuracy	0.037	0.032	0.032	0.996	0.319	-5.950

Plots of demographic effects from ODI-based FI Mapping

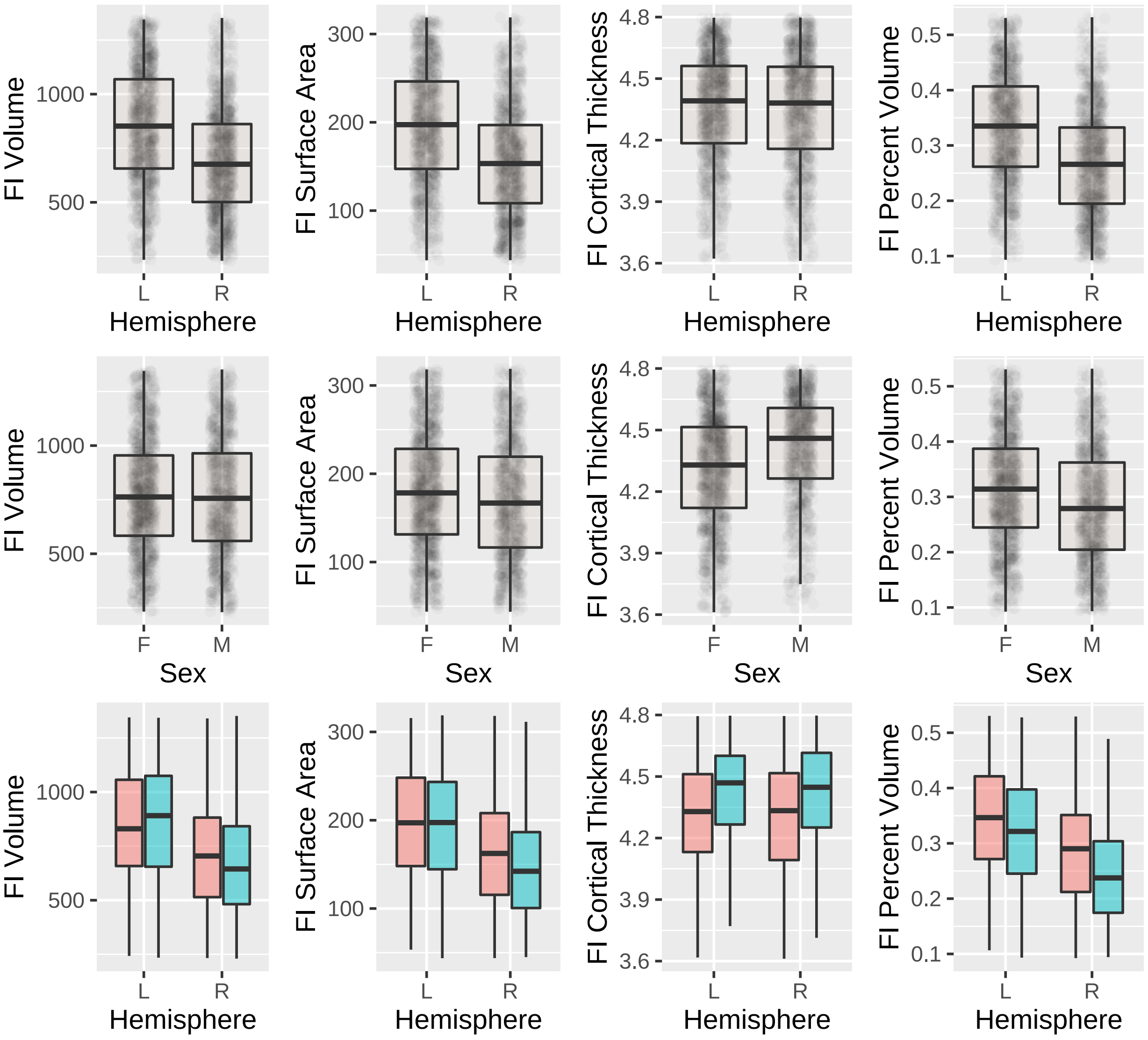

Plots of demographic effects from FA-based FI Mapping

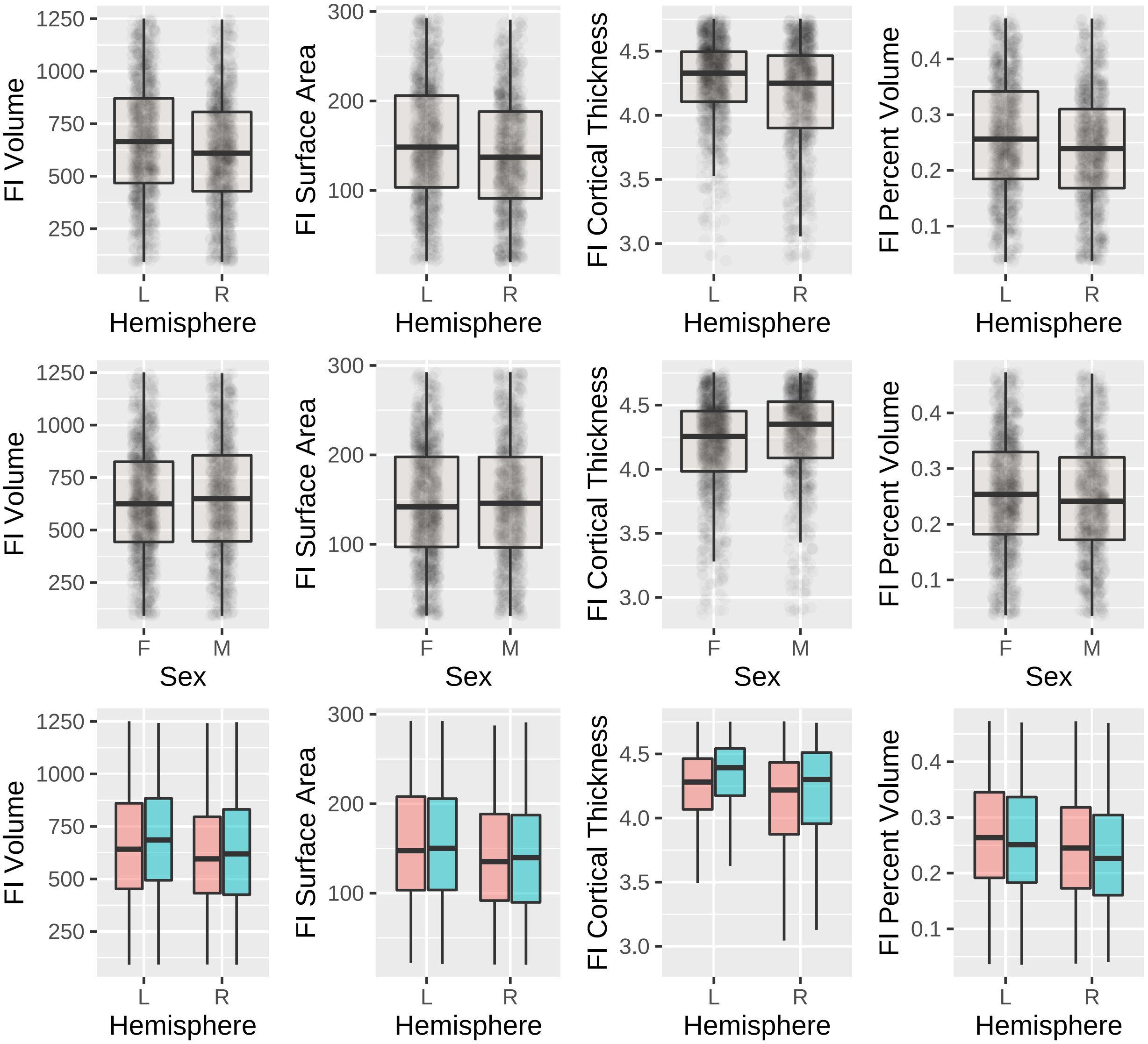

Plots of demographic effects from whole cortical volume

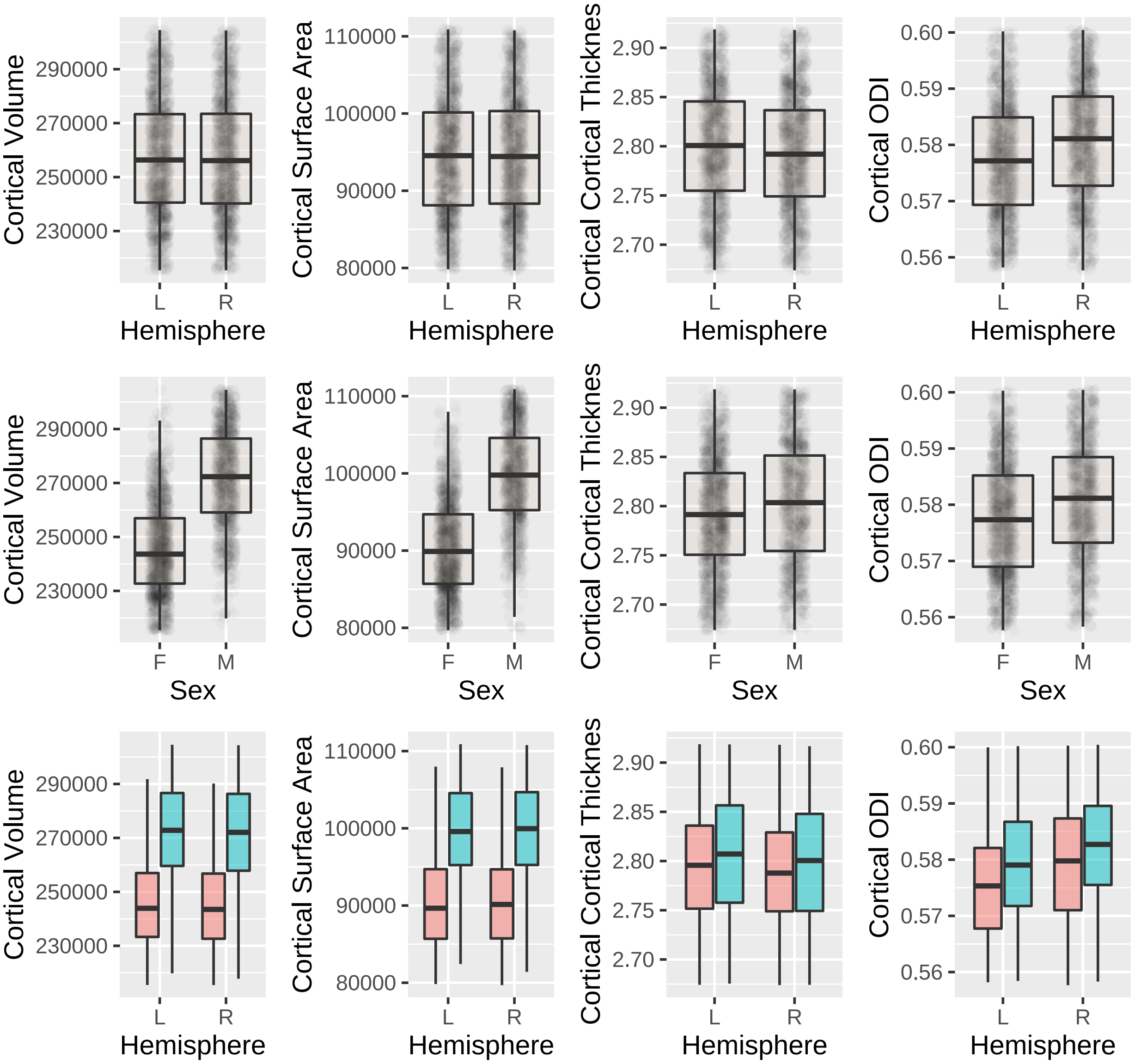

Plots of behavioral variables from ODI-based FI mapping

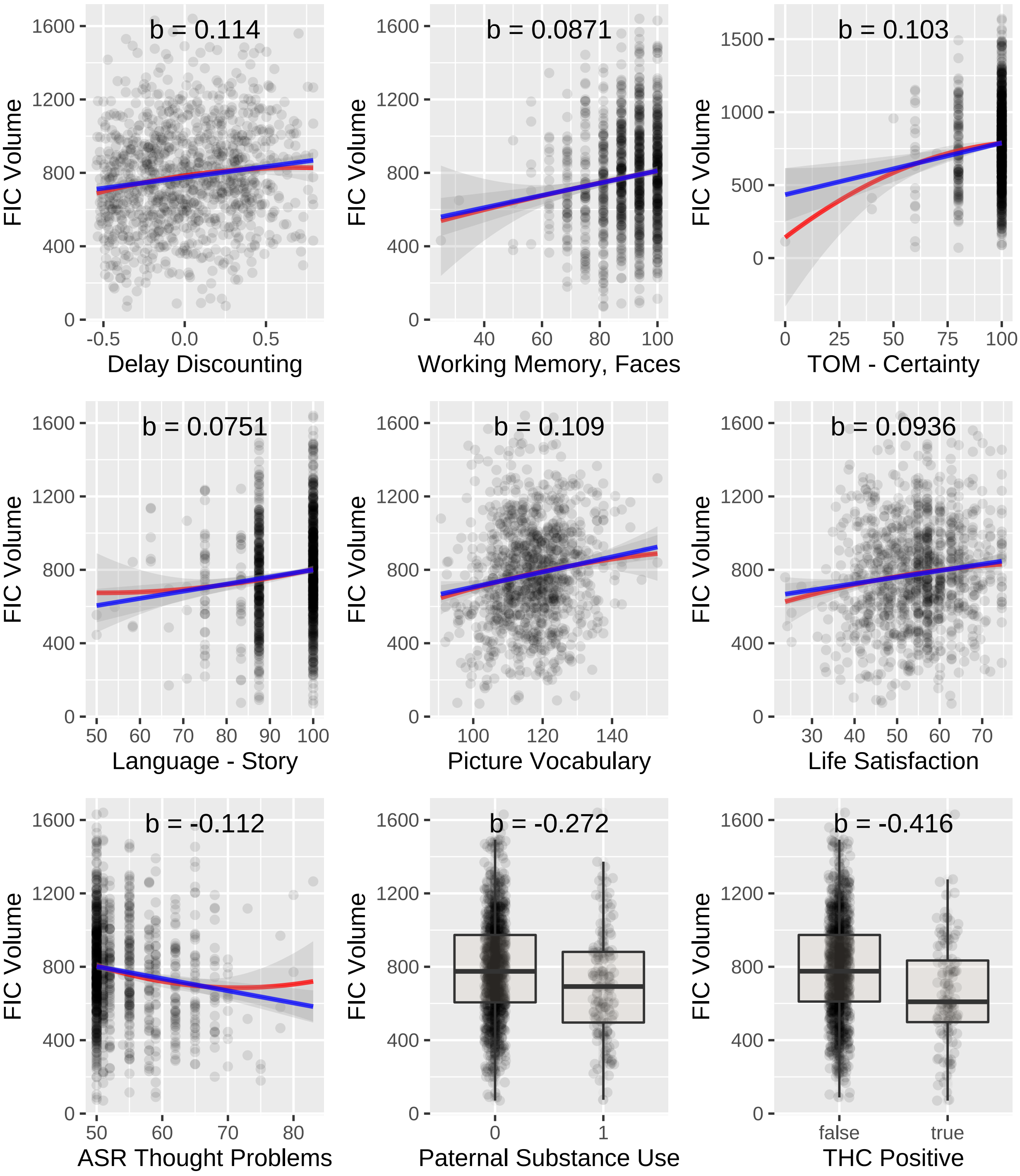

Plots of behavioral variables from FA-based FI mapping

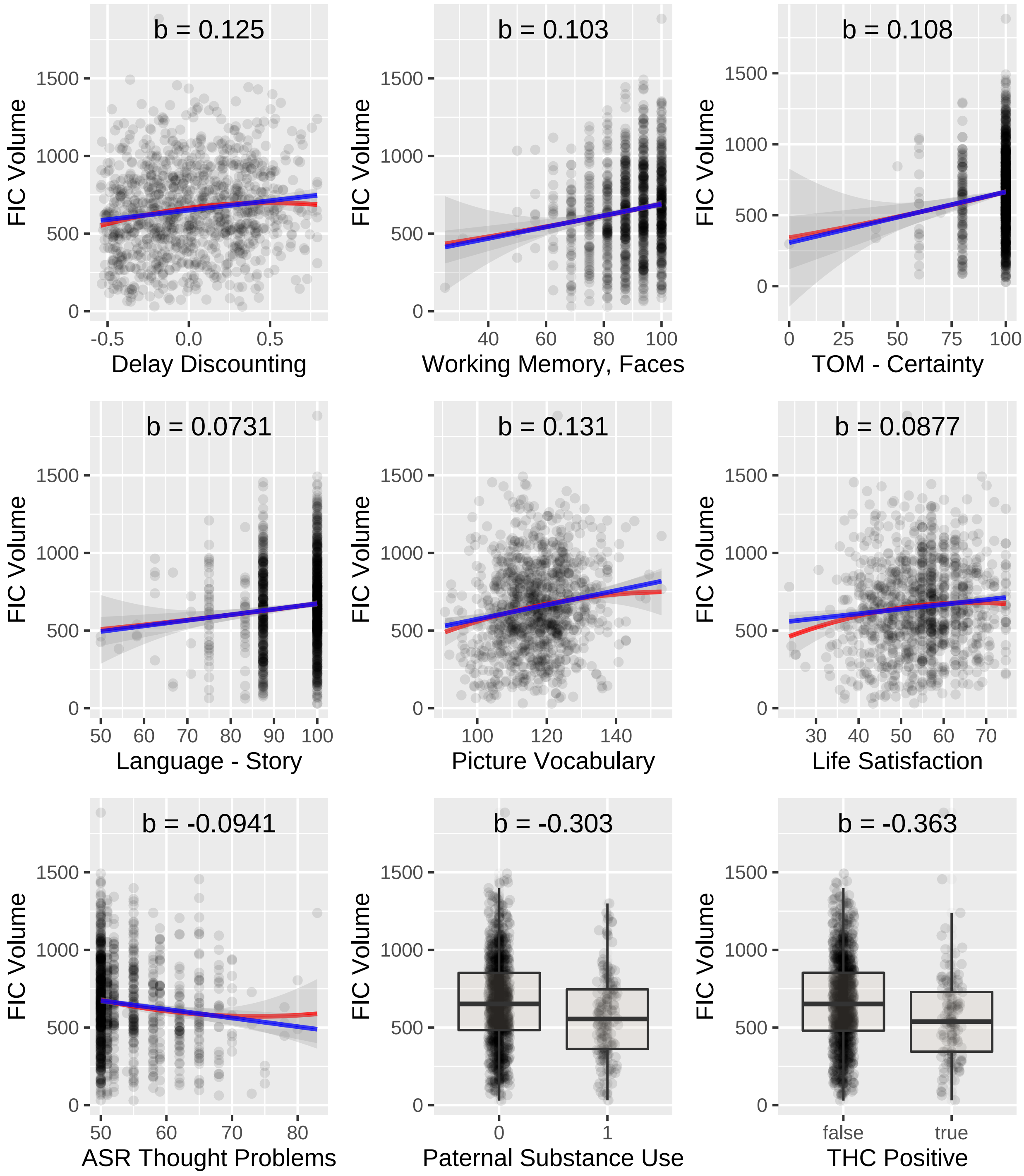

Reliability and Heritability from ODI-based FI mapping

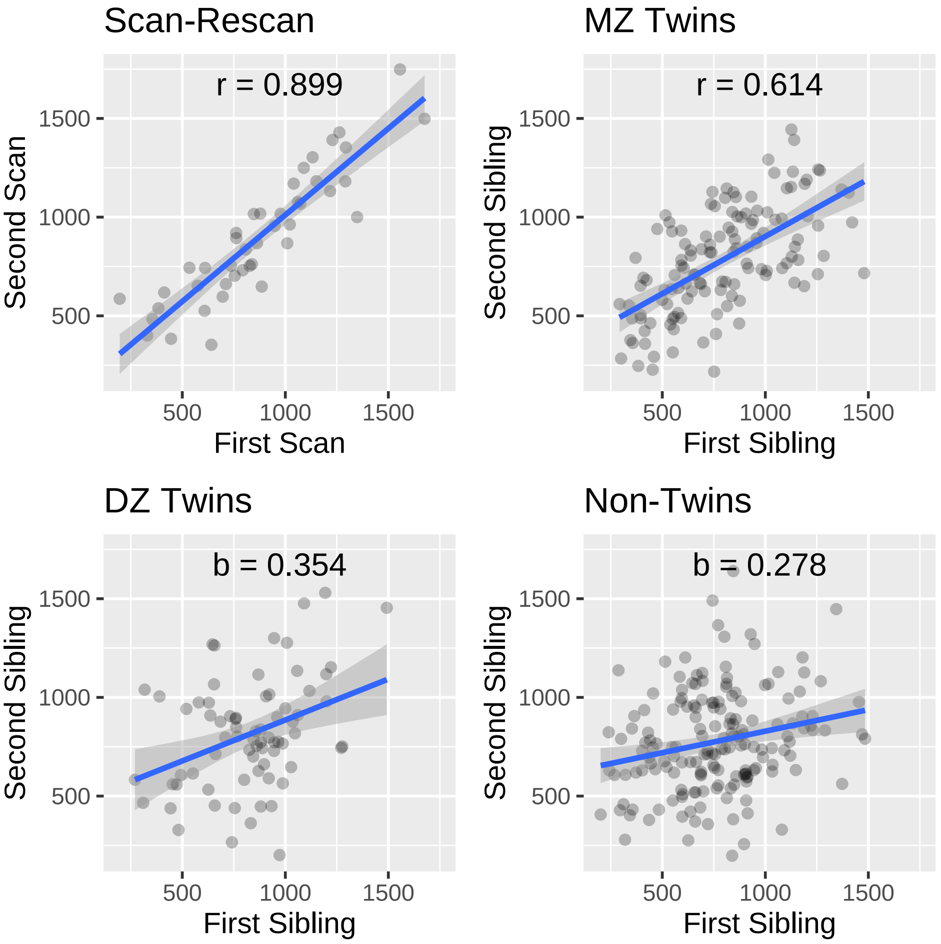

Table 14: Test-Retest and Heritability of FI volume derived from diffusion microstructure

Name	Value
Individual Test-Retest Correlation	0.899
Monozygotic Twin Pair Correlation	0.614
Dizygotic Twin Pair Correlation	0.354
Non-Twin Sibling Pair Correlation	0.278
Falconer's Heritability Score	0.521

Reliability and Heritability from FA-based FI mapping

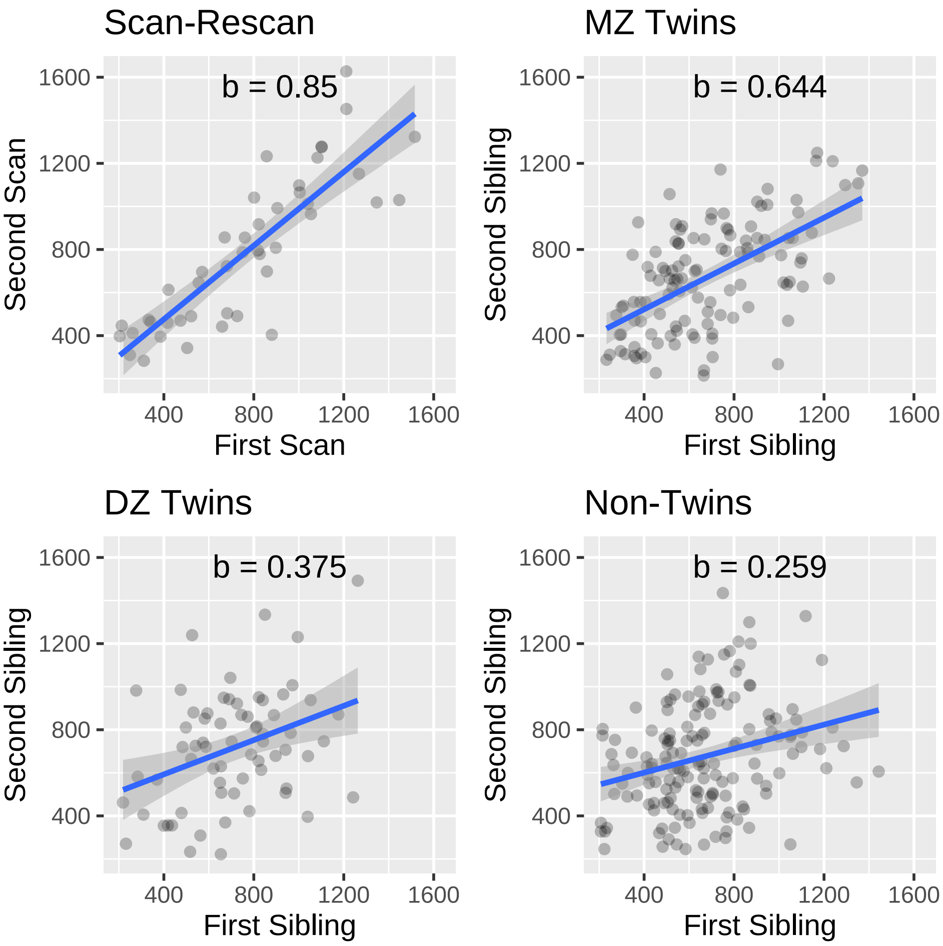

Table 15: Test-Retest and Heritability of FI volume derived from diffusion microstructure

Name	Value
Individual Test-Retest Correlation	0.850
Monozygotic Twin Pair Correlation	0.644
Dizygotic Twin Pair Correlation	0.375
Non-Twin Sibling Pair Correlation	0.259
Falconer's Heritability Score	0.538

Reliability and Heritability AAIC Region Volume

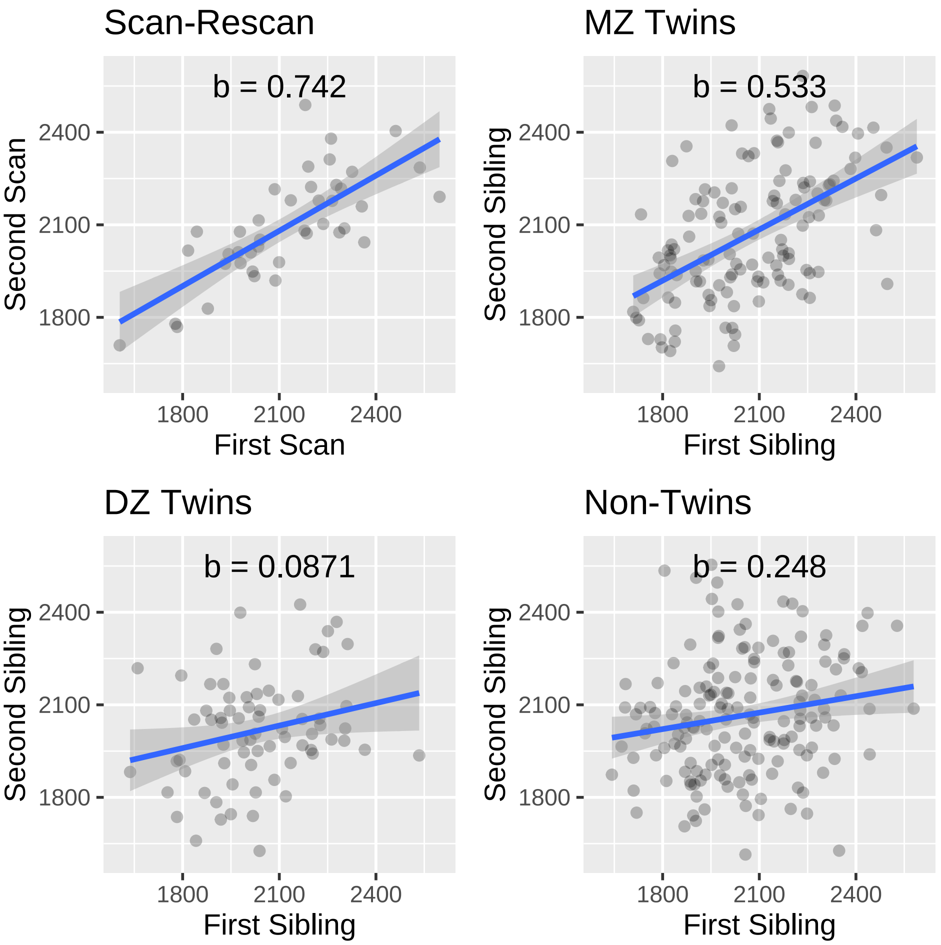

Table 16: Test-Retest and Heritability of Freesurfer-derived AAIC volume

Name	Value
Individual Test-Retest Correlation	0.742
Monozygotic Twin Pair Correlation	0.533
Dizygotic Twin Pair Correlation	0.087
Non-Twin Sibling Pair Correlation	0.248
Falconer's Heritability Score	0.893

Reliability and Heritability from Whole Cortex Volume

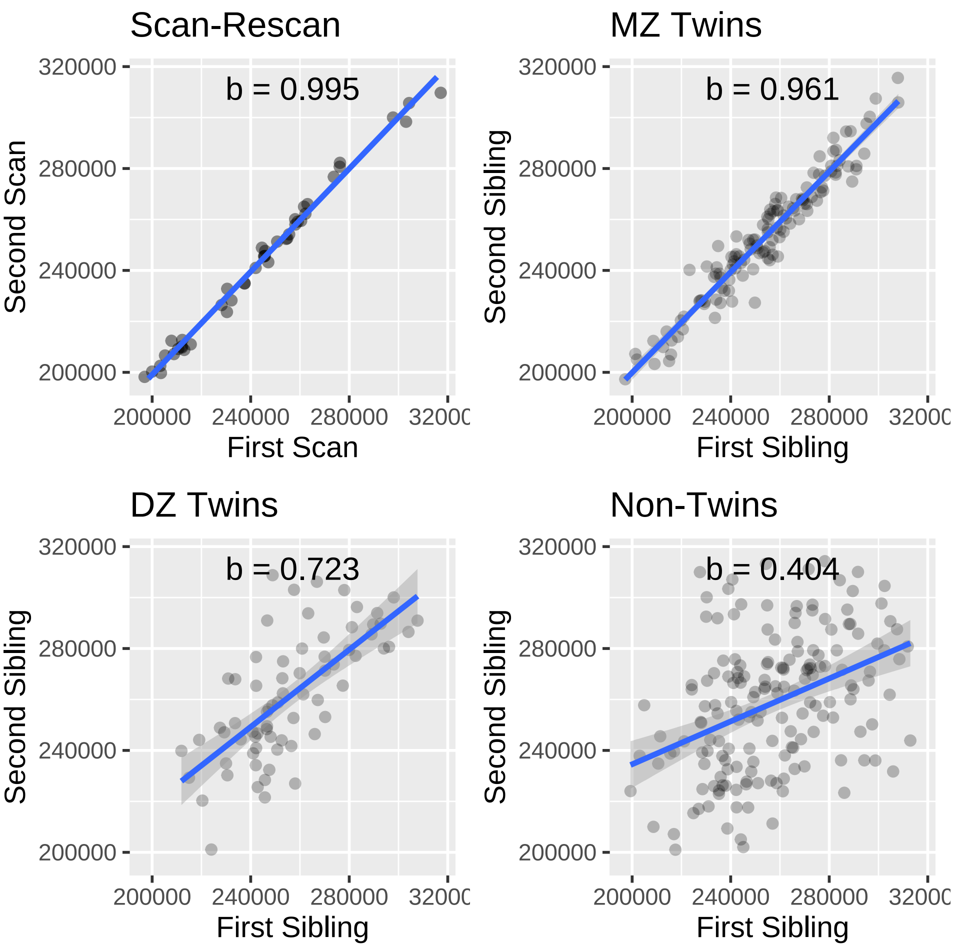

Table 17: Test-Retest and Heritability of FI volume derived from diffusion microstructure

Name	Value
Individual Test-Retest Correlation	0.995
Monozygotic Twin Pair Correlation	0.961
Dizygotic Twin Pair Correlation	0.723
Non-Twin Sibling Pair Correlation	0.404
Falconer's Heritability Score	0.476
